# Estimating Lower Limb Kinematics Using a Lie Group Constrained Extended Kalman Filter with a Reduced Wearable IMU Count and Distance Measurements †

**DOI:** 10.3390/s20236829

**Published:** 2020-11-29

**Authors:** Luke Wicent F. Sy, Nigel H. Lovell, Stephen J. Redmond

**Affiliations:** 1 Graduate School of Biomedical Engineering, UNSW Sydney, Sydney 2052, Australia; n.lovell@unsw.edu.au; 2 UCD School of Electrical and Electronic Engineering, University College Dublin, Belfield, 4 Dublin, Ireland; stephen.redmond@ucd.ie

**Keywords:** Lie group, constrained extended Kalman filter, gait analysis, motion capture, pose estimation, wearable devices, IMU, distance measurement

## Abstract

Tracking the kinematics of human movement usually requires the use of equipment that constrains the user within a room (e.g., optical motion capture systems), or requires the use of a conspicuous body-worn measurement system (e.g., inertial measurement units (IMUs) attached to each body segment). This paper presents a novel Lie group constrained extended Kalman filter to estimate lower limb kinematics using IMU and inter-IMU distance measurements in a reduced sensor count configuration. The algorithm iterates through the prediction (kinematic equations), measurement (pelvis height assumption/inter-IMU distance measurements, zero velocity update for feet/ankles, flat-floor assumption for feet/ankles, and covariance limiter), and constraint update (formulation of hinged knee joints and ball-and-socket hip joints). The knee and hip joint angle root-mean-square errors in the sagittal plane for straight walking were $7.6\pm 2.6^{\circ }$ and $6.6\pm 2.7^{\circ }$, respectively, while the correlation coefficients were 0.95±0.03 and 0.87±0.16, respectively. Furthermore, experiments using simulated inter-IMU distance measurements show that performance improved substantially for dynamic movements, even at large noise levels (σ=0.2 m). However, further validation is recommended with actual distance measurement sensors, such as ultra-wideband ranging sensors.

## 1. Introduction

Human pose estimation is the tracking of position and orientation (i.e., pose) of body segments. Joint angles can then be calculated from the relative pose of linked body segments. Applications exist in robotics, virtual reality, animation, and healthcare (e.g., gait analysis). Traditionally, human pose is captured within a laboratory setting using optical motion capture (OMC) systems with up to millimeter level position accuracy [1] when properly configured and calibrated. However, the setup for OMC systems is time consuming and inconvenient (e.g., multiple markers are taped to the body) and requires considerable expertise. Recent miniaturization of inertial measurements units (IMUs) has paved the path toward inertial motion capture (IMC) systems suitable for prolonged use outside of the laboratory. Some examples of clinical applications in the current literature include determining level of spinal damage [2] and Parkinson’s disease diagnosis [3]. Furthermore, developing a comfortable IMC for routine daily use may facilitate interactive rehabilitation [4,5], and allow the study of movement disorder progression to enable predictive diagnostics.

Commercial IMCs attach one sensor per body segment (OSPS) [6], which may be considered too cumbersome and expensive for routine daily use by a consumer due to the number of IMUs required. Similar works also exist in the literature using quaternions [7], Kalman filters (KF) [8], and particle filters [9]. Each IMU typically tracks the orientation of the attached body segment using an orientation estimation algorithm (e.g., [10,11]), which is then connected via linked kinematic chain, usually rooted at the pelvis. A reduced-sensor-count (RSC) configuration, where IMUs are placed on a subset of body segments, can improve user comfort, reduce setup time and system cost. However, using fewer wearable sensor units necessarily reduces the kinematic information available, which must otherwise be inferred from (i) our knowledge of human movement (e.g., enforcing mechanical joint constraints or making dynamic balance assumptions), or (ii) by using additional sensing modalities within each wearable sensor unit. Each approach will be described in the next subsections.

### 1.1. Existing Algorithms for Human Motion Tracking Using Fewer IMUs

The performance of algorithms with RSC configuration depends (i) on how the kinematic information of uninstrumented body segments is inferred and (ii) on how body pose is represented.

The kinematic information of body segments which do not have sensors attached to them may be inferred by the algorithm, either through data obtained in the past (i.e., observed correlations between co-movement of different body segments or data-driven approaches) or by using a simplified kinematic model of the human body (i.e., model-based approaches). Data-driven approaches (e.g., nearest-neighbor search [12] and bi-directional recurrent neural network [13]) are able to recreate realistic motion suitable for animation-related applications. However, these approaches are normally biased toward motions already contained in the database, which may limit their use in monitoring pathological gait. Model-based approaches reconstruct human movement using kinematic and biomechanical models (e.g., linear regression [14], constrained KF [15], extended KF (EKF) [16], particle filter [9], and window-based optimization [17]). The use of optimization-based estimators is sometimes favoured due to its relative ease to setup and ease of understanding. However, the algorithm can be very inefficient when tracking a larger number of dimensions (e.g., when tracking body pose over a long duration). To significantly increase the efficiency of the algorithm when estimating body pose across time, a recursive estimator can take advantage of the state substructure and reduce the state dimension being tracked [18].

Body poses are usually represented using Euler angles or quaternions [9,16]. However, recent work on pose estimation has shown that using a Lie group to represent the states of recursive estimator is a promising approach. Such algorithms typically represent the body pose as a chain of linked segments using matrix Lie groups to represent the orientation or pose of each body segment; specifically the special orthogonal group, SO(n), and special Euclidean group, SE(n), where n=2,3, are the spatial dimensions for humam body kinematics problems. The early works of Wang et al. [19] and Barfoot et al. [20] investigated representations and propagation of pose uncertainty, the former in the context of manipulator kinematics and the latter focused on SE(3), followed by the formulation of recursive estimators using Lie group representation (e.g., EKF [21] and unscented KF (UKF) [22]). Recent literature has reported the use of Lie group based recursive estimators to estimate human movement. Cesic et al. estimated the full body pose from OMC marker measurements and achieved significant improvements compared to an Euler angle representation [23]; and even supplemented the approach with an observability analysis [24]. Joukov et al. represented the human body using SO(n), tracking the pose using measurements from IMUs under an OSPS configuration [25]. Joukov et al.’s algorithm was tested using an arm tracking experiment, where the results improved especially during arm poses that cause a singularity when using an Euler angle representation (i.e., Lie group representation is singularity free).

### 1.2. Improving Human Pose Estimation Using Additional Sensor Measurements

Another approach is to supplement kinematic information from the IMU with another kind of sensor, which inherently increases cost and reduces battery life. Note that we will focus on systems that supplement pose estimation, not on the global position estimation of the subject (e.g., [26]). For example, IMCs can be supplemented with standard video cameras (e.g., fused using an optimization-based algorithm [27], and deep neural networks [28]) or depth cameras [29] at fixed locations in the capture environment, external to the subject. The combination of IMCs and portable cameras solves a weakness of OMCs (i.e., marker or body segment occlusion) and a weakness of IMCs (i.e., global position drift). However, the system still requires an external sensor that is carried by another person or requires some quick setup. IMCs can also be supplemented by distance measurements (using ultrasonic devices and KF in OSPS configuration [30], using constrained KF in RSC configuration [31]), removing dependence on any external non-body-worn sensor.

### 1.3. Novelty

This paper describes a novel human pose estimator that represents the state using Lie groups with the state propagated iteratively using a constrained EKF (CEKF) to estimate lower body kinematics for an RSC configuration of IMUs and inter-IMU distance measurements; the Lie group framework and inclusion of inter-IMU distance measurements, along with the exploration of its effect on pose estimation accuracy, are the major advancements made in this paper. It extends the work of [32] and builds on prior work of [15,31], but instead represents the state variables as elements of Lie groups, specifically SE(3), to track both position and orientation (whereas [15] only tracks position and assumes orientation measurements are noise-free). Furthermore, this paper presents in detail a novel Lie group formulation for assumptions typically used in human pose estimation (e.g., zero velocity update, constant body segment lengths, and a hinged knee joint). While not our focus here, our algorithm, with its SE(3) representation, is able to track the global position of body segments, taking into account IMU measurements during the prediction step, and a simpler implementation of certain measurement assumptions (e.g., zero velocity update), though at the expense of having an additional constraint step to ensure satisfaction of biomechanical constraints. The algorithm design was motivated by the need for a body pose representation that more closely models the human biomechanical system (without a dramatic increase in the dimensions of the tracked state) from which the missing kinematic information of uninstrumented body segments are inferred. The contributions of this paper advance the development of gait assessment tools for comfortable and long-term monitoring of lower body movement.

## 2. Mathematical Background: Lie Group and Lie Algebra

The matrix Lie group *G* is a group of n×n matrices (e.g., SE(3)). Mathematically speaking, it is also a smooth manifold with smooth group composition and inversion (i.e., matrix multiplication and inversion). The Lie algebra g can be represented in the vector space and is closely related to Lie group *G*. It represents a tangent space of a group at the identity element [33]. The elegance of Lie theory lies in its ability to represent pose using a vector space (e.g., Lie group *G* is represented by g) without additional constraints (e.g., without requiring $R^{T}R=I$ which is using a rotation matrix representation of orientation, or ||q||=1 which is using a quaternion representation of orientation) [34].

**Figure 1:**
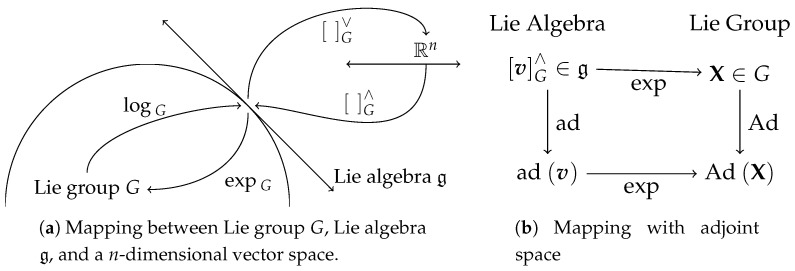
Overview of Lie group theory mappings. When G=SE(3), Lie group X=T is a 4×4 transformation matrix representing pose (i.e., 3D rotation and translation). Similarly, v=ξ where Lie algebra ${\left[\xi \right]}_{SE(3)}^{\wedge }\in \mathrm{se}\left(3\right)$ and the vector $\xi \in R^{n}$ with n=6.

The matrix exponential $\exp{}_{G}:g\to G$ (Equation (1)) and matrix logarithm $\log{}_{G}:G\to g$ relates (i.e., local diffeomorphism) the Lie group *G* and its Lie algebra g. The Lie algebra g is a n×n matrix that can be represented compactly in an *n*-dimensional vector space. A linear isomorphism (i.e., one-to-one mapping) between g and $R^{n}$ is given by operators ${\left[\;\;\right]}_{G}^{\vee }:g\to R^{n}$ and ${\left[\;\;\right]}_{G}^{\wedge }:R^{n}\to g$, which map between the compact and matrix representation of the Lie algebra g. Figure 1 shows an illustration of the said mappings.

Furthermore, the adjoint operator of a Lie group, ${\mathrm{Ad}}_{G}\left(X\right)$, the adjoint operator of a Lie algebra, ${\mathrm{ad}}_{G}\left(v\right)$, and the right jacobian, $\Phi_{G}\left(v\right)$ (Equation (2)), where X∈G and ${\left[v\right]}_{G}^{\wedge }\in g$ will be used in later sections. Multiplying an *n*-dimensional vector representation of a Lie algebra with ${\mathrm{Ad}}_{G}\left(X\right)\in R^{n\times n}$ (i.e., the product ${\mathrm{Ad}}_{G}\left(X\right)v$) transforms the vector from one coordinate frame to another, similar to how rotation matrices transform points from one frame to another. ${\mathrm{ad}}_{G}\left(v\right)$ is the Lie algebra of ${\mathrm{Ad}}_{G}\left(X\right)$.A summary of the operators for Lie groups SO(3), SE(3), and $R^{n}$ will be explained in the next subsections. They will serve as building blocks to implement the algorithm being described by this paper. For a more detailed introduction to Lie groups refer to [18,34,35].

(1) $$\begin{array}{c} \exp\left({\left[v\right]}_{G}^{\wedge }\right)=\sum_{n=0}^{\infty }\frac{1}{n!}{\left({\left[v\right]}_{G}^{\wedge }\right)}^{n} \end{array}$$

(2) $$\begin{array}{c} \Phi_{G}\left(v\right)=\sum_{i=0}^{\infty }\frac{{\left(-1\right)}^{i}}{(i+1)!}{\mathrm{ad}}_{G}{\left(v\right)}^{i}\;,\;v\in R^{n} \end{array}$$

### 2.1. Special Orthogonal Group SO(3)

The special orthogonal group, $SO\left(3\right):=\left\{R\in R^{3\times 3}|RR^{T}=1,\det R=1\right\}$, represents orientation, where R is the typical 3×3 rotation matrix whose column vectors represent the *x*, *y*, and *z* basis vectors. The operations for SO(3) are listed below, and will serve as building blocks for SE(3), which will be described in the next subsection. Note that ${\left[a\right]}_{SO(3)}^{\wedge }b$ is equivalent to the cross product of a and b. See Chapter 7 of [18] for details.

(3) $$\begin{array}{c} {\left[\phi \right]}_{SO(3)}^{\wedge }={\left[\begin{array}{c} \phi_{1}\\ \phi_{2}\\ \phi_{3} \end{array}\right]}_{SO(3)}^{\wedge }=\left[\begin{array}{ccc} 0 & -\phi_{3} & \phi_{2}\\ \phi_{3} & 0 & -\phi_{1}\\ -\phi_{2} & \phi_{1} & 0 \end{array}\right],\;{\left[\begin{array}{ccc} 0 & -\phi_{3} & \phi_{2}\\ \phi_{3} & 0 & -\phi_{1}\\ -\phi_{2} & \phi_{1} & 0 \end{array}\right]}_{SO(3)}^{\vee }=\left[\begin{array}{c} \phi_{1}\\ \phi_{2}\\ \phi_{3} \end{array}\right]=\phi \end{array}$$

If ϕ/|ϕ| represents a unit vector axis we wish to rotate around, and |ϕ| is the angle by which we wish to rotate, then the rotation matrix, R, which will implement this rotation is given by Equation (4), which is also known as the Rodrigues’ axis-angle rotation formula. When ϕ is very small, $R\approx I_{3\times 3}+{\left[\phi \right]}_{SO(3)}^{\wedge }$.

(4) $$\begin{array}{c} R=\exp\left({\left[\phi \right]}_{SO(3)}^{\wedge }\right)=\cos\left(|\phi |\right)I_{3\times 3}+\left(1-\cos\left(|\phi |\right)\right)\frac{\phi \phi^{T}}{{\left|\phi \right|}^{2}}+\sin\left(|\phi |\right){\left[\frac{\phi }{\left|\phi \right|}\right]}_{SO(3)}^{\wedge } \end{array}$$

Furthermore, the Lie algebra adjoint, Lie group adjoint, and inverse operators are listed in Equation (5).

(5) $$\begin{array}{c} {\mathrm{ad}}_{SO(3)}\left(\phi \right)={\left[\phi \right]}_{SO(3)}^{\wedge },\;{\mathrm{Ad}}_{SO(3)}\left(R\right)=R,\;R^{-1}=R^{T} \end{array}$$

Lastly, to approximate the compound rotations, $R_{1}R_{2}$, in the Lie algebra space where $R_{1}=\;\exp\left({\left[\phi_{1}\right]}_{SO(3)}^{\wedge }\right)$ and $R_{2}=\exp\left({\left[\phi_{2}\right]}_{SO(3)}^{\wedge }\right)$, we can use Equation (6). The right Jacobian, $\Phi_{SO(3)}\left(\phi \right)\in R^{3\times 3}$, is obtained using Equation (7).

(6) $$\begin{array}{c} {\left[\log\left(R_{1}R_{2}\right)\right]}_{SO(3)}^{\vee }\approx \phi_{1}+\Phi_{SO(3)}{\left(\phi_{1}\right)}^{-1}\phi_{2}\in \mathrm{so}\left(3\right) \end{array}$$

(7) $$\begin{array}{c} \Phi_{SO(3)}\left(\phi \right)=\frac{\sin(|\phi |)}{\left|\phi \right|}I_{3\times 3}+\left(1-\frac{\sin(|\phi |)}{\left|\phi \right|}\right)\frac{\phi \phi^{T}}{{\left|\phi \right|}^{2}}-\frac{1-\cos(|\phi |)}{\left|\phi \right|}{\left[\frac{\phi }{\left|\phi \right|}\right]}_{SO(3)}^{\wedge }\in R^{3\times 3} \end{array}$$

### 2.2. Special Euclidean Group, SE(3)

The special Euclidean group, $SE\left(3\right):=\left\{T=\left[\begin{array}{cc} R & t\\ 0^{T} & 1 \end{array}\right]\in R^{4\times 4}|\left\{R,t\right\}\in SO\left(3\right)\times R^{3}\right\}$, represents orientation and translation, where T is the typical 4×4 transformation matrix, R is the rotation matrix, and t represents a coordinate point in Euclidean space. The operations for SE(3) are listed below. $I_{i\times i}$ and $0_{i\times j}$ denote i×i identity and i×j zero matrices. See Chapter 7 of [18] for details.

(8) $$\begin{array}{c} {\left[\xi \right]}_{SE(3)}^{\wedge }={\left[\begin{array}{c} \rho \\ \phi \end{array}\right]}_{SE(3)}^{\wedge }=\left[\begin{array}{cc} {\left[\phi \right]}_{SO(3)}^{\wedge } & \rho \\ 0_{1\times 3} & 0 \end{array}\right],\;{\left[\begin{array}{cc} {\left[\phi \right]}_{SO(3)}^{\wedge } & \rho \\ 0_{1\times 3} & 0 \end{array}\right]}_{SE(3)}^{\vee }=\left[\begin{array}{c} \rho \\ \phi \end{array}\right] \end{array}$$

(9) $$\begin{array}{c} T=\exp\left({\left[\xi \right]}_{SE(3)}^{\wedge }\right)=\left[\begin{array}{cc} \exp({\left[\phi \right]}_{SO(3)}^{\wedge }) & \Phi_{SO(3)}\left(-\phi \right)\rho \\ 0_{1\times 3} & 1 \end{array}\right]=\left[\begin{array}{cc} R & t\\ 0_{1\times 3} & 1 \end{array}\right] \end{array}$$

(10) $$\begin{array}{c} {\mathrm{ad}}_{SE(3)}\left(\xi \right)=\left[\begin{array}{cc} {\left[\phi \right]}_{SO(3)}^{\wedge } & {\left[\rho \right]}_{SO(3)}^{\wedge }\\ 0_{3\times 3} & {\left[\phi \right]}_{SO(3)}^{\wedge } \end{array}\right],\;\;{\mathrm{Ad}}_{SE(3)}\left(T\right)=\left[\begin{array}{cc} R & {\left[\rho \right]}_{SO(3)}^{\wedge }R\\ 0_{3\times 3} & R \end{array}\right],\;\;T^{-1}=\left[\begin{array}{cc} R^{T} & -R^{T}\rho \\ 0_{1\times 3} & 1 \end{array}\right] \end{array}$$

Lastly, we note the useful identity defined in Equation (11) where ${\left[a\right]}_{SE(3)}^{\wedge },{\left[b\right]}_{SE(3)}^{\wedge }\in \mathrm{se}\left(3\right)$ which is the Lie algebra of the Lie Group SE(3) ([18], Equation (72)), which will be used to compute the Jacobians of our model later.

(11) $$\begin{array}{c} {\left[a_{}^{}\right]}_{SE(3)}^{\wedge }b={\left[b\right]}_{SE(3)}^{⊙}a,\;\mathrm{where}\;b=\left[\begin{array}{c} \epsilon \\ \eta \end{array}\right],\;{\left[b\right]}_{}^{⊙}=\left[\begin{array}{cc} \eta I_{3\times 3} & -{\left[\epsilon \right]}_{SO(3)}^{\wedge }\\ 0_{1\times 3} & 0_{1\times 3} \end{array}\right],\;\epsilon \in R^{3},\;\eta \in R \end{array}$$

### 2.3. Real Numbers $R^{n}$

In order to represent vector state variables (e.g., translation, velocity, and acceleration) and be consistent with how we used SE(3) to represent pose, we represented the real numbers $s\in R^{n}$ as SE(n) poses with position and no rotation. The operations for $R^{n}$ are listed below.

(12) $$\begin{array}{c} {\left[s\right]}_{R^{n}}^{\wedge }=\left[\begin{array}{cc} 0_{n\times n} & s\\ 0_{1\times n} & 0 \end{array}\right],\;{\left[\begin{array}{cc} 0_{n\times n} & s\\ 0_{1\times n} & 0 \end{array}\right]}_{R^{n}}^{\vee }=s \end{array}$$

(13) $$\begin{array}{c} S=\exp\left({\left[s\right]}_{R^{n}}^{\wedge }\right)=\left[\begin{array}{cc} I_{n\times n} & s\\ 0_{1\times n} & 1 \end{array}\right],\;{\left[\log\left(\left[\begin{array}{cc} I_{n\times n} & s\\ 0_{1\times n} & 1 \end{array}\right]\right)\right]}_{R^{n}}^{\vee }=s,\;\exp{\left({\left[s\right]}_{R^{n}}^{\wedge }\right)}^{-1}=\left[\begin{array}{cc} I_{n\times n} & -s\\ 0_{1\times n} & 1 \end{array}\right] \end{array}$$

(14) $$\begin{array}{c} {\mathrm{ad}}_{R^{n}}\left(s\right)=0_{n\times n},\;{\mathrm{Ad}}_{R^{n}}\left(S\right)=I_{n\times n},\;\Phi_{R^{n}}\left(s\right)=0_{n\times n} \end{array}$$

Note that the multiplication of two elements of the Lie group (i.e., the exponential of $s_{1}$ and $s_{2}$) is equivalent to the vector addition of two elements of the Lie algebra (i.e., $s_{1}+s_{2}$).

(15) $$\begin{array}{c} {\left[\log\left(\exp\left({\left[s_{1}\right]}_{R^{n}}^{\wedge }\right)\exp\left({\left[s_{2}\right]}_{R^{n}}^{\wedge }\right)\right)\right]}_{R^{n}}^{\vee }=s_{1}+s_{2} \end{array}$$

## 3. Algorithm Description

**Figure 2:**
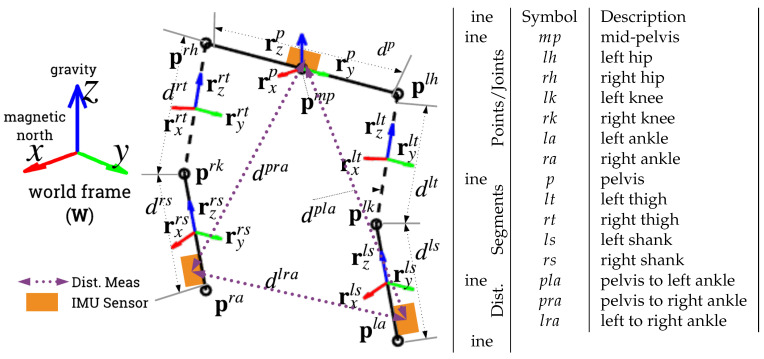
Model of the lower body used by LGKF-3IMU. The circles denote joint positions, the solid lines denote instrumented body segments, whilst the dashed lines denote segments without IMUs attached (i.e., thighs). Dotted lines denote inter-IMU measurements.

**Figure 3:**
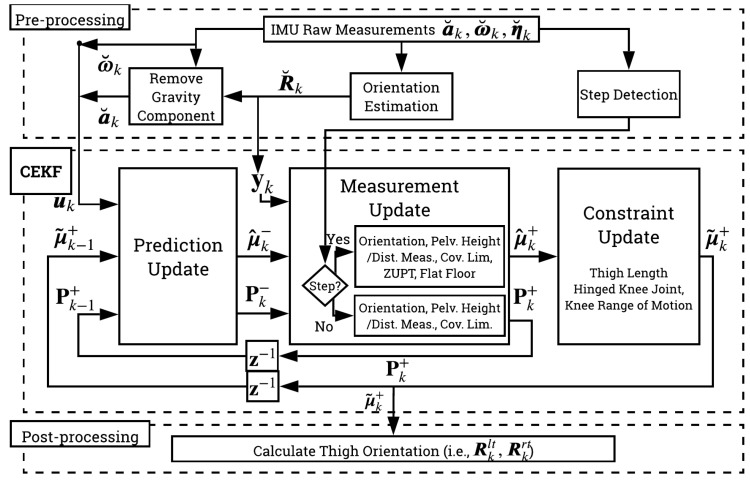
Algorithm overview which consists of pre-processing, CEKF, and post-processing. Pre-processing calculates the body segment orientation, inertial body acceleration (calculated by expressing IMU acceleration with respect world frame using its current orientation estimate then subtracting gravity), and step detection from raw acceleration, ${\overset{˘}{a}}_{k}$, angular velocity, ${\overset{˘}{\omega }}_{k}$, and magnetic north heading, ${\overset{˘}{\eta }}_{k}$, measured by the IMU. The CEKF-based state estimation consists of a prediction (kinematic equation), measurement (orientation, pelvis height/inter-IMU distance measurement, covariance limiter, intermittent zero-velocity update, and flat-floor assumption), and constraint update (thigh length, hinge knee joint, and knee range of motion). Post-processing calculates the left and right thigh orientations, $R_{k}^{lt}$ and $R_{k}^{rt}$.

The proposed algorithm, L5S-3IMU, uses a similar model and assumptions to our prior works in [15,31], denoted as CKF-3IMU and CKF-3IMU+D, albeit expressed in Lie group representation. The algorithm uses measurements from three IMUs attached at the pelvis (sacrum) and shanks (slightly above the ankles), and the inter-IMU distance measurements to estimate the orientation of five body segments (i.e., pelvis, thighs, and shanks) with respect the world frame, *W* (Figure 2). The Lie group representation enables the tracking of both position and orientation (note that CKF-3IMU only tracked position and assumed orientation measurements were noise-free). Figure 3 shows an overview of the proposed algorithm. L5S-3IMU predicts the ankle and pelvis positions through double integration of their linear 3D acceleration, and predicts the shank and pelvis orientation through integration of their linear 3D angular velocity. The orientation estimates are further updated using a third-party orientation estimation algorithm. Drift in the position estimates due to sensor noise, which accumulates quadratically in the double integration of acceleration, was mitigated through the following assumptions: (1) the pelvis position is approximated as the height of the pelvis when the leg(s) are unbent, or as informed by inter-IMU distance measurements, when available; and (2) the ankle velocity and height above the floor are zeroed whenever a footstep is detected. Furthermore, a pseudo-measurement equal to the current global position estimate of the pelvis and ankles is made with a fixed covariance to contain the ever-growing error covariance of the said states. Lastly, constant body segment lengths and hinged knee joints (one degree of freedom (DOF)) with limited range of motion (ROM) were enforced as biomechanical constraints. The pre- and post-processing parts remain exactly the same as the CKF-3IMU algorithm (e.g., acceleration due to body movement was calculated by expressing the acceleration of the instrumented body segment in the world frame using the orientation estimate and then subtracting acceleration due to gravity (i.e., $g={\left[0\;\;0\;\;9.81\right]}^{T}$) [6]).

### 3.1. System, Measurement, and Constraint Models

The system, measurement, and constraint models are presented below

(16) $$\begin{array}{c} X_{k}=f\left(X_{k-1},u_{k},n_{k}\right)=X_{k-1}\exp\left({\left[\Omega \left(X_{k-1},u_{k}\right)+n_{k}\right]}_{G}^{\wedge }\right) \end{array}$$

(17) $$\begin{array}{c} Z_{k}=h\left(X_{k}\right)\exp\left({\left[m_{k}\right]}_{G}^{\wedge }\right),\;D_{k}=c\left(X_{k}\right) \end{array}$$

where *k* is the time step. $X_{k}\in G$ is the system state, an element of state Lie group *G*. $\Omega \left(X_{k-1},u_{k}\right):G\to R^{p}$ is a non-linear function which describes how the model acts on the state and input, $u_{k-1}$, where *p* is the number of dimensions of the compact vector representation for Lie algebra g. $n_{k}$ is a zero-mean process noise vector with covariance matrix Q (i.e., $n_{k}∼N_{R^{p}}\left(0_{p\times 1},Q\right)$). $Z_{k}\in G_{m}$ is the system measurement, an element of the measurement Lie group $G_{m}$. $h\left(X_{k}\right):G\to G_{m}$ is the measurement function. $m_{k}$ is a zero-mean measurement noise vector with covariance matrix $R_{k}$ (i.e., $m_{k}∼N_{R^{q}}\left(0_{q\times 1},R_{k}\right)$ where *q* is the number of dimensions of available measurements). $D_{k}\in G_{c}$ is the constraint state, an element of constraint Lie group $G_{c}$. $c\left(X_{k}\right):G\to G_{c}$ is the equality constraint function the state $X_{k}$ must satisfy (i.e., $c\left(X_{k}\right)=D_{k}$). Similar to [23,36], the state distribution of $X_{k}$ is assumed to be a concentrated Gaussian distribution on Lie groups (i.e., $X_{k}=\mu_{k}\exp_{G}{\left[\epsilon \right]}_{G}^{\wedge }$, where $\mu_{k}$ is the mean of $X_{k}$ and Lie algebra error $\epsilon ∼N_{R^{p}}\left(0_{p\times 1},P\right)$) [19].

The Lie group state variables $X_{k}$ model the position, orientation, and velocity of the three instrumented body segments (i.e., pelvis and shanks) as $X_{k}=\mathrm{diag}\left(T_{}^{p},T_{}^{ls},T_{}^{rs},\exp\left({\left[{\left[{\left(v_{}^{p}\right)}^{T}\;{\left(v_{}^{ls}\right)}^{T}\;{\left(v_{}^{rs}\right)}^{T}\right]}^{T}\right]}_{R^{9}}^{\wedge }\right)\right)$∈$G=SE{\left(3\right)}^{3}\times R^{9}$ where $T_{}^{b}\in SE\left(3\right)$ represents the pose (i.e., orientation and position) of body segment *b* relative to world frame *W*, and ${vA}_{}^{b}$ is the velocity of body segment *b* relative to frame *A*. If frame *A* is not specified, assume reference to the world frame, *W*. The Lie algebra error is denoted as $\epsilon ={\left[{\left(\epsilon_{T}^{p}\right)}^{T}\;\;{\left(\epsilon_{T}^{ls}\right)}^{T}\;\;{\left(\epsilon_{T}^{rs}\right)}^{T}\;\;{\left(\epsilon_{v}^{mp}\right)}^{T}\;\;{\left(\epsilon_{v}^{la}\right)}^{T}\;\;{\left(\epsilon_{v}^{ra}\right)}^{T}\right]}^{T}$ where the first three variables correspond to the Lie group in SE(3) while the latter three are for $R^{9}$. ${\left[\;\right]}_{G}^{\vee }$, $\exp({\left[\;\right]}_{G}^{\wedge })$, ${\left[\log\left(\;\right)\right]}_{G}^{\vee }$, ${\mathrm{Ad}}_{G}\left(X_{k}\right)$, and $\Phi_{G}\left(\;\right)$ are constructed similarly as $X_{k}$(e.g., ${\mathrm{Ad}}_{G}\left(X_{k}\right)=\mathrm{diag}\left({\mathrm{Ad}}_{SE(3)}\left(T_{}^{p}\right),{\mathrm{Ad}}_{SE(3)}\left(T_{}^{ls}\right),{\mathrm{Ad}}_{SE(3)}\left(T_{}^{rs}\right),{\mathrm{Ad}}_{R^{9}}\left(\exp\left({\left[{\left[{\left(v_{}^{p}\right)}^{T}\;{\left(v_{}^{ls}\right)}^{T}\;{\left(v_{}^{rs}\right)}^{T}\right]}^{T}\right]}_{R^{9}}^{\wedge }\right)\right)\right)$. Refer to Section 2.2 and Section 2.3 for definition of SE(3) and $R^{n}$ operators.

### 3.2. Lie Group Constrained EKF (LG-CEKF)

The a priori, a posteriori, and constrained state estimate (i.e., estimated mean of $X_{k}$) for time step *k* are denoted by ${\hat{\mu }}_{k}^{-}$, ${\hat{\mu }}_{k}^{+}$, and ${\tilde{\mu }}_{k}^{+}$, respectively. Note that the true state $X_{k}$ can be expressed as $\mu_{k}\exp\left({\left[\epsilon \right]}_{G}^{\wedge }\right)$ where $\mu_{k}$ is one of the state means just mentioned with error, ${\left[\epsilon \right]}_{G}^{\wedge }$. The a priori and a posteriori error covariance matrices are denoted as $P_{k}^{-}$ and $P_{k}^{+}$, respectively. Note the error covariance is not updated at the constrain update step. The KF is based on the Lie group EKF, as defined in [36], where the state means (${\hat{\mu }}_{k}^{-}$, ${\hat{\mu }}_{k}^{+}$, and ${\tilde{\mu }}_{k}^{+}$) and state error covariance matrices ($P_{k}^{-}$ and $P_{k}^{+}$) are propagated by the KF at each time step.

#### 3.2.1. Prediction Step

Prediction step estimates the a priori state ${\hat{\mu }}_{k}^{-}$ at the next time step. The mean propagation of the three instrumented body segments is governed by Equation (18) where $\Omega ({\tilde{\mu }}_{k-1}^{+},u_{k})$ (Equation (19)) is the motion model for the three instrumented body segments. Note that the state propagation may not necessarily respect the biomechanical constraints, so joints may become dislocated after this step. The input $u_{k}$ is defined in Equation (20), where the orientation and acceleration as obtained by the IMU attached to segment *b* with respect world frame *W* are denoted as ${\overset{˘}{R}}_{k}^{b}$ and ${\overset{˘}{a}}_{k}^{b}$ for b∈{p,ls,rs}, while the angular velocity as obtained by the IMU attached to segment *b* expressed in frame *b* is denoted as ${\overset{˘}{\omega }b}_{k}^{}$.

(18) $$\begin{array}{c} {\hat{\mu }}_{k}^{-}={\tilde{\mu }}_{k-1}^{+}\exp\left({\left[\Omega \left({\tilde{\mu }}_{k-1}^{+},u_{k}\right)\right]}_{G}^{\wedge }\right) \end{array}$$

(19) $$\begin{array}{c} \begin{array}{cc} \Omega \left({\tilde{\mu }}_{k-1}^{+},u_{k}\right) & =\underset{1\times 3}{\underset{︸}{\begin{array}{c} {}[{\left(\Delta t{\tilde{v}}_{k-1}^{mp+}+\frac{\Delta t^{2}}{2}{\overset{˘}{a}}_{k}^{p}\right)}^{T}{\overset{˘}{R}}_{k}^{p} \end{array}}}\;\underset{1\times 3}{\underset{︸}{\begin{array}{c} \Delta t{\overset{˘}{\omega }p}_{k}^{T} \end{array}}}\;\underset{1\times 3}{\underset{︸}{\begin{array}{c} {\left(\Delta t{\tilde{v}}_{k-1}^{la+}+\frac{\Delta t^{2}}{2}{\overset{˘}{a}}_{k}^{ls}\right)}^{T}{\overset{˘}{R}}_{k}^{ls} \end{array}}}\;\underset{1\times 3}{\underset{︸}{\begin{array}{c} \Delta t{\overset{˘}{\omega }ls}_{k}^{T} \end{array}}}\\ & \underset{1\times 3}{\underset{︸}{\begin{array}{c} {\left(\Delta t{\tilde{v}}_{k-1}^{ra+}+\frac{\Delta t^{2}}{2}{\overset{˘}{a}}_{k}^{rs}\right)}^{T}{\overset{˘}{R}}_{k}^{rs} \end{array}}}\;\underset{1\times 3}{\underset{︸}{\begin{array}{c} \Delta t{\overset{˘}{\omega }rs}_{k}^{T} \end{array}}}\;\underset{1\times 9}{\underset{︸}{\begin{array}{c} \Delta t{\left({\overset{˘}{a}}_{k}^{mp}\right)}^{T}\;\Delta t{\left({\overset{˘}{a}}_{k}^{la}\right)}^{T}\;\Delta t{\left({\overset{˘}{a}}_{k}^{ra}\right)}^{T}{]}^{T} \end{array}}} \end{array} \end{array}$$

(20) $$\begin{array}{c} u_{k}=\left[\begin{array}{ccccccccc} {\overset{˘}{R}}_{k}^{p} & {\overset{˘}{R}}_{k}^{ls} & {\overset{˘}{R}}_{k}^{rs} & {\overset{˘}{a}}_{k}^{p} & {\overset{˘}{a}}_{k}^{ls} & {\overset{˘}{a}}_{k}^{rs} & {\overset{˘}{\omega }p}_{k}^{} & {\overset{˘}{\omega }ls}_{k}^{} & {\overset{˘}{\omega }rs}_{k}^{} \end{array}\right] \end{array}$$

The state error covariance matrix propagation is governed by Equation (21), where $F_{k}$ represents the matrix Lie group equivalent to the Jacobian of $f(X_{k-1},n_{k-1})$, Q is the covariance matrix of the process noise, and $C_{k}=\frac{\partial }{\partial \epsilon }\Omega \left({\tilde{\mu }}_{k-1}^{+}\exp\left({\left[\epsilon \right]}_{G}^{\wedge }\right),u_{k}\right){|}_{\epsilon =0}$ represents the linearization of the motion model with an infinitesimal perturbation ϵ. The process noise covariance matrix, Q, is calculated from the input-to-state matrix G and the noise variances of the measured acceleration and angular velocity, $\sigma_{a}^{2}$ and $\sigma_{\omega }^{2}$, respectively.

(21) $$\begin{array}{c} P_{k}^{-}=F_{k}P_{k-1}^{+}F_{k}^{T}+\Phi_{G}\left(\Omega \left({\tilde{\mu }}_{k-1}^{+},u_{k}\right)\right)Q\Phi_{G}{\left(\Omega \left({\tilde{\mu }}_{k-1}^{+},u_{k}\right)\right)}^{T} \end{array}$$

(22) $$\begin{array}{c} F_{k}={\mathrm{Ad}}_{G}\left(\exp_{G}\left(-{\left[\Omega \left({\tilde{\mu }}_{k-1}^{+},u_{k}\right)\right]}_{G}^{\wedge }\right)\right)+\Phi_{G}\left(\Omega \left({\tilde{\mu }}_{k-1}^{+},u_{k}\right)\right)C_{k} \end{array}$$

(23) $$\begin{array}{c} Q=G\;\mathrm{diag}\left(\sigma_{a}^{2},\sigma_{\omega }^{2}\right)\;G^{T} \end{array}$$

(24)
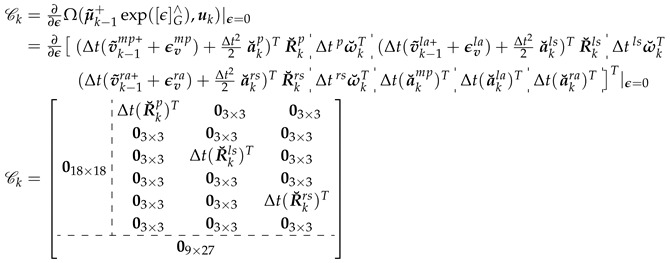

(25) $$\begin{array}{c} G=\left[\begin{array}{cccccc} \Delta t^{2}\;/\;2\;I_{3\times 3} & 0_{3\times 3} & 0_{3\times 3} & 0_{3\times 3} & 0_{3\times 3} & 0_{3\times 3}\\ 0_{3\times 3} & 0_{3\times 3} & 0_{3\times 3} & \Delta tI_{3\times 3} & 0_{3\times 3} & 0_{3\times 3}\\ 0_{3\times 3} & \Delta t^{2}\;/\;2\;I_{3\times 3} & 0_{3\times 3} & 0_{3\times 3} & 0_{3\times 3} & 0_{3\times 3}\\ 0_{3\times 3} & 0_{3\times 3} & 0_{3\times 3} & 0_{3\times 3} & \Delta tI_{3\times 3} & 0_{3\times 3}\\ 0_{3\times 3} & 0_{3\times 3} & \Delta t^{2}\;/\;2\;I_{3\times 3} & 0_{3\times 3} & 0_{3\times 3} & 0_{3\times 3}\\ 0_{3\times 3} & 0_{3\times 3} & 0_{3\times 3} & 0_{3\times 3} & 0_{3\times 3} & \Delta tI_{3\times 3}\\ & \Delta tI_{9\times 9} & & & 0_{9\times 9} & \end{array}\right] \end{array}$$

#### 3.2.2. Measurement Update

Measurement update estimates the state at the next time step by: (i) updating the orientation state using new orientation measurements of body segments from IMUs; by (ii) encouraging pelvis position to be above the feet, as informed by either some pseudo-measurement or inter-IMU distance measurements; and by (iii) enforcing ankle velocity to reach zero, and the ankle *z* position to be near the floor level, $z_{f}$ when step is detected. When only IMU measurements are available, (iia) pelvis *z* position is encouraged to be close to initial standing height, $z_{p}$. When inter-IMU distance measurements are available, (iia) is not used. Instead, (iib) ankle distance is directly incorporated while pelvis position is inferred from inter-IMU distance measurements assuming hinged knee joints and constant body segment lengths. The a posteriori state mean ${\hat{\mu }}_{k}^{+}$ is calculated following the Lie EKF equations below. Note that ${\left[\log\left(h{\left({\hat{\mu }}_{k}^{-}\right)}^{-1}Z_{k}\right)\right]}_{G_{m}}^{\vee }$ in Equation (27) is akin to the KF innovation/residual, where $h{\left({\hat{\mu }}_{k}^{-}\right)}^{-1}Z_{k}$ (derived from Equation (17) assuming $m_{k}=0$ and $X_{k}={\hat{\mu }}_{k}^{-}$, i.e., $Z_{k}=h\left({\hat{\mu }}_{k}^{-}\right)$) is the innovation/residual in Lie group $G_{m}$ brought to the vector representation of the Lie algebra space using the inverse exponential (i.e., logarithm) mapping.

(26) $$\begin{array}{c} {\hat{\mu }}_{k}^{+}={\hat{\mu }}_{k}^{-}\exp_{G}\left({\left[\nu_{k}\right]}_{G}^{\wedge }\right) \end{array}$$

(27) $$\begin{array}{c} \nu_{k}=K_{k}\left({\left[\log\left(h{\left({\hat{\mu }}_{k}^{-}\right)}^{-1}Z_{k}\right)\right]}_{G_{m}}^{\vee }\right) \end{array}$$

(28) $$\begin{array}{c} K_{k}=P_{k}^{-}H_{k}^{T}{\left(H_{k}P_{k}^{-}H_{k}^{T}+R_{k}\right)}^{-1} \end{array}$$

$H_{k}$ can be seen as the matrix Lie group equivalent to the Jacobian of $h(X_{k})$, and is defined as the concatenation of $H_{ori}$ and $H_{mp,k}$ when inter-IMU distance measurement is not available. When inter-IMU distance measurement is available, $H_{mp,k}$ is replaced by $H_{dist,k}={\left[H_{pla,k}^{T}\;H_{pra,k}^{T}\;H_{lra,k}^{T}\right]}^{T}$. $H_{ls,k}$ and/or $H_{rs,k}$ are also concatenated to $H_{k}$ when the left and/or right foot contact (FC) is detected (See Equation (9) of [15]). Each component matrix will be described later. The measurement matrix $Z_{k}\in G_{m}$, measurement function $h\left(X_{k}\right)\in G_{m}$, and measurement covariance noise $R_{k}$ are constructed similarly to $H_{k}$, but combined using diag instead of concatenation (e.g., $R_{k}=\mathrm{diag}\left(\sigma_{ori}^{2},\sigma_{mp}^{2}\right)$).

(29) $$\begin{array}{c} \begin{array}{cc} H_{k} & =\frac{\partial }{\partial \epsilon }{\left[\log\left(h{\left({\hat{\mu }}_{k}^{-}\right)}^{-1}h\left({\hat{\mu }}_{k}^{-}\exp\left({\left[\epsilon \right]}_{G}^{\wedge }\right)\right)\right)\right]}_{G_{m}}^{\vee }{|}_{\epsilon =0}\\ & =\left\{\begin{array}{cc} {\left[H_{ori}^{T}\;H_{mp/dist}^{T}\right]}^{T} & \;\mathrm{no}\;\mathrm{FC}\\ {}[H_{ori}^{T}\;H_{mp/dist}^{T}\;H_{ls,k}^{T}{]}^{T} & \;\mathrm{left}\;\mathrm{FC}\\ {}[H_{ori}^{T}\;H_{mp/dist}^{T}\;H_{rs,k}^{T}{]}^{T} & \;\mathrm{right}\;\mathrm{FC}\\ {}[H_{ori}^{T}\;H_{mp/dist}^{T}\;H_{ls,k}^{T}\;H_{rs,k}^{T}{]}^{T} & \;\mathrm{both}\;\mathrm{FC} \end{array}\right. \end{array} \end{array}$$

##### Orientation Update

The orientation update utilizes the orientation measurement to update the state estimate as defined by Equation (30), with measurement noise variance $\sigma_{ori}^{2}$ (9×1 vector).

(30) $$\begin{array}{c} h_{ori}\left(X_{k}\right)=\mathrm{diag}\left(R_{k}^{p},R_{k}^{ls},R_{k}^{rs}\right)\in SO{\left(3\right)}^{3},\;Z_{ori}=\mathrm{diag}\left({\overset{˘}{R}}_{k}^{p},{\overset{˘}{R}}_{k}^{ls},{\overset{˘}{R}}_{k}^{rs}\right) \end{array}$$

$H_{ori}$ along with other components of $H_{k}$ are calculated by applying Equation (29) to their corresponding measurement functions, followed by tedious algebraic manipulation and first order linearization (i.e., $\exp\left({\left[\epsilon \right]}_{}^{\wedge }\right)\approx I+{\left[\epsilon \right]}_{}^{\wedge }$). The derivation for $H_{ori}$ (Equation (31)) can be solved trivially as ${\left[\log\left(h_{ori}{\left({\hat{\mu }}_{k}^{-}\right)}^{-1}h_{ori}\left({\hat{\mu }}_{k}^{-}\exp\left({\left[\epsilon \right]}_{G}^{\wedge }\right)\right)\right)\right]}_{}^{\vee }={\left[{\left(\epsilon_{\phi }^{p}\right)}^{T}\;\;{\left(\epsilon_{\phi }^{ls}\right)}^{T}\;\;{\left(\epsilon_{\phi }^{rs}\right)}^{T}\right]}^{T}$, where $\epsilon_{T}^{b}={\left[{\left(\epsilon_{\rho }^{b}\right)}^{T}\;\;{\left(\epsilon_{\phi }^{b}\right)}^{T}\right]}^{T}$ for body segment b∈{p,ls,rs}.

(31) $$\begin{array}{c} H_{ori}=\left[\begin{array}{cccc} 0_{3\times 3}\;I_{3\times 3} & & & \\ & 0_{3\times 3}\;I_{3\times 3} & & 0_{9\times 9}\\ & & 0_{3\times 3}\;I_{3\times 3} & \end{array}\right] \end{array}$$

##### Pelvis Height Assumption

The pelvis height assumption softly constrains the pelvis *z* position to be close to initial standing height $z_{p}$ as defined by Equation (32) (represented in vector space of its Lie algebra) and Equation (33), with measurement noise variance $\sigma_{mp}^{2}$ (1×1 vector). This assumption is used only when inter-IMU distance measurement is not available. $i_{x}$, $i_{y}$, $i_{z}$, and $i_{0}$ denote 4×1 vectors whose 1st to 4th rows, respectively, are 1, while the rest are 0; they are used below to select rows, columns, or elements from matrices.

(32) $$\begin{array}{c} {\left[\log\left(h_{mp}^{}\left(X_{k}\right)\right)\right]}_{}^{\vee }=i_{z}^{T}T_{k}^{p}i_{0}=\left[\begin{array}{cccc} 0 & 0 & 1 & 0 \end{array}\right]\left[\begin{array}{cc} R_{k}^{p} & p_{k}^{p}\\ 0_{1\times 3} & 1 \end{array}\right]\left[\begin{array}{c} 0\\ 0\\ 0\\ 1 \end{array}\right]=\left[\begin{array}{cccc} 0 & 0 & 1 & 0 \end{array}\right]\left[\begin{array}{c} p_{k}^{p}\\ 1 \end{array}\right]=p_{z,k}^{p}\in R \end{array}$$

(33) $$\begin{array}{c} \log\left(Z_{mp}^{}\right){]}_{}^{\vee }=z_{p}\in R \end{array}$$

The derivation of $H_{mp,k}=\frac{\partial }{\partial \epsilon }{\left[\log\left(h_{mp}^{}{\left({\hat{\mu }}_{k}^{-}\right)}^{-1}h_{mp}^{}\left({\hat{\mu }}_{k}^{-}\exp\left({\left[\epsilon \right]}_{G}^{\wedge }\right)\right)\right)\right]}_{}^{\vee }{|}_{\epsilon =0}$ is shown in Equations (34)–(36). Taking best estimate $X_{k}={\hat{\mu }}_{k}^{-}$ gives us Equation (34).

(34) $$\begin{array}{c} {\left[\log\left(h_{mp}^{}\left({\hat{\mu }}_{k}^{-}\right)\right)\right]}_{}^{\vee }=i_{z}^{T}{\hat{T}}_{k}^{p-}i_{0} \end{array}$$

(35) $$\begin{array}{c} \begin{array}{cc} {\left[\log\left(h_{mp}^{}\left({\hat{\mu }}_{k}^{-}\exp\left({\left[\epsilon \right]}_{G}^{\wedge }\right)\right)\right)\right]}_{}^{\vee } & =i_{z}^{T}{\hat{T}}_{k}^{p-}\exp\left({\left[\epsilon_{T}^{p}\right]}_{}^{\wedge }\right)i_{0}\\ & \approx i_{z}^{T}{\hat{T}}_{k}^{p-}i_{0}+i_{z}^{T}{\hat{T}}_{k}^{p-}{\left[\epsilon_{T}^{p}\right]}_{}^{\wedge }i_{0},\;1\mathrm{st}\;\mathrm{order}\;\mathrm{linearization}\\ & \mathrm{Use}\;\mathrm{Equation}\;(11),\;{\left[a_{}^{}\right]}_{}^{\wedge }b_{}^{}={\left[b_{}^{}\right]}_{}^{⊙}a_{}^{},\;\mathrm{to}\;\mathrm{bring}\;\epsilon_{T}^{p}\;\mathrm{to}\;\mathrm{right}\;\mathrm{of}\;i_{0}\\ & ={\left[\log\left(h_{mp}^{}\left({\hat{\mu }}_{k}^{-}\right)\right)\right]}_{}^{\vee }+i_{z}^{T}{\hat{T}}_{k}^{p-}{\left[i_{0}\right]}_{}^{⊙}\epsilon_{T}^{p} \end{array} \end{array}$$

Remember $\epsilon_{T}^{p}$ is a subvector of $\epsilon_{}^{}$ as defined in Section 3.1 and is the Lie algebra error of the state in its compact vector representation. Note that if measurement function $h_{a}^{}\left(X_{k}\right)\in$ Lie group $R^{b}$, then ${\left[\log\left(h_{a}^{}{\left({\hat{\mu }}_{k}^{-}\right)}^{-1}h_{a}^{}\left(X_{k}\right)\right)\right]}_{}^{\vee }={\left[\log\left(h_{a}^{}\left(X_{k}\right)\right)\right]}_{}^{\vee }-{\left[\log\left(h_{a}^{}\left({\hat{\mu }}_{k}^{-}\right)\right)\right]}_{}^{\vee }={\left[\log\left(h_{a}^{}\left({\hat{\mu }}_{k}^{-}\exp\left({\left[\epsilon \right]}_{G}^{\wedge }\right)\right)\right)\right]}_{}^{\vee }-{\left[\log\left(h_{a}^{}\left({\hat{\mu }}_{k}^{-}\right)\right)\right]}_{}^{\vee }$ by applying Equations (15) and (13) (inverse of Lie group $R^{n}$). Finally, $H_{mp,k}$ is calculated as shown in Equation (36).
(36)
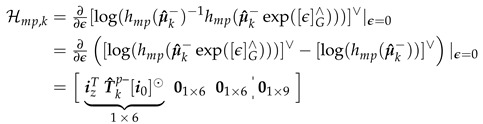


##### Zero Velocity Update and Flat Floor Assumption

When step is detected, the ankle velocity is enforced to be zero and the ankle *z* position is brought to near the floor level, $z_{f}$ (i.e., flat floor assumptions). The corresponding measurement function is defined by Equation (37), with measurement noise variance $\sigma_{ls}^{2}$ (4×1 vector).

(37) $$\begin{array}{c} {\left[\log\left(h_{ls}^{}\left(X_{k}\right)\right)\right]}_{}^{\vee }=\left[\begin{array}{c} v_{}^{ls}\\ i_{z}^{T}T_{k}^{ls}i_{0} \end{array}\right]=\left[\begin{array}{c} v_{}^{ls}\\ p_{z,k}^{ls} \end{array}\right]\in R^{4},\;{\left[\log\left(Z_{ls}^{}\right)\right]}_{}^{\vee }=\left[\begin{array}{c} 0_{3\times 1}\\ z_{f} \end{array}\right] \end{array}$$

The zero velocity part of $H_{ls,k}$ (Equation (38)) and $H_{rs,k}$ can also be calculated trivially, while the flat floor assumption can be calculated similarly as $H_{mp,k}$ but the *z* position set to floor height, $z_{f}$, instead of the pelvis standing height, $z_{p}$.
(38)
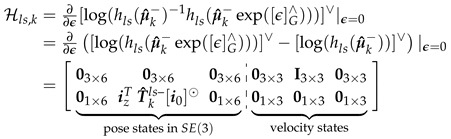


##### Left and Right Ankle Distance Measurement

When the inter-IMU distance between the ankles, ${\overset{˘}{d}}_{k}^{lra}$, is available, ankle distance measurement is incorporated as a soft distance constraint. The measurement function is defined by Equation (40), with measurement noise variance $\sigma_{lra}^{2}$ (1×1 vector). $\tau_{lra}^{}\left(X_{k}\right)$ (Equation (39)) is the vector that points from the right ankle to the left ankle, where ${pls}_{}^{la}$ is the position of the left ankle expressed in left shank frame, and ${prs}_{}^{ra}$ is the position of the right ankle expressed in right shank frame. We have chosen that the ankles are at the origin of their respective shank frames. Note that matrix E converts homogeneous 4×1 coordinates to standard 3×1 coordinates (i.e., drops the 1 from the end of the 4×1 vector).

(39) $$\begin{array}{c} \tau_{lra}^{}\left(X_{k}\right)=\overset{E}{\overset{︷}{\left[\begin{array}{cc} I_{3\times 3} & 0_{3\times 1} \end{array}\right]}}\left(\overset{\mathrm{left}\;\mathrm{ankle}\;\mathrm{in}\;W}{\overset{︷}{T_{k}^{ls}\;{pls}_{}^{la}}}-\overset{\mathrm{right}\;\mathrm{ankle}\;\mathrm{in}\;W}{\overset{︷}{T_{k}^{rs}\;{prs}_{}^{ra}}}\right),\;{pls}_{}^{la}={prs}_{}^{ra}=\overset{\mathrm{origin}\;\mathrm{of}\;\mathrm{frame}}{\overset{︷}{{\left[\begin{array}{cccc} 0 & 0 & 0 & 1 \end{array}\right]}^{T}}} \end{array}$$

By taking the squared Euclidean distance of $\tau_{lra}^{}\left(X_{k}\right)$ (i.e., $\left|\right|\tau_{lra}^{}\left(X_{k}\right){\left|\right|}^{2}$), we can get the ankle distance measurement model.

(40) $$\begin{array}{c} {\left[\log\left(h_{lra}^{}\left(X_{k}\right)\right)\right]}_{}^{\vee }={\left(\tau_{lra}^{}\left(X_{k}\right)\right)}^{T}\tau_{lra}^{}\left(X_{k}\right)\in R,\;{\left[\log\left(Z_{lra}^{}\right)\right]}_{}^{\vee }={\left({\overset{˘}{d}}_{k}^{lra}\right)}^{2} \end{array}$$

To solve for $H_{lra,k}$ (Equation (44)), we first solved for ${\left[\log\left(h_{lra}^{}\left(X_{k}\right)\right)\right]}_{}^{\vee }$ at $X_{k}={\hat{\mu }}_{k}^{-}$ (Equation (41)).

(41) $$\begin{array}{c} \tau_{lra}^{}\left({\hat{\mu }}_{k}^{-}\right)=E\left({\hat{T}}_{k}^{ls-}\;{pls}_{}^{la}-{\hat{T}}_{k}^{rs-}\;{prs}_{}^{ra}\right),\;{\left[\log\left(h_{lra}^{}\left({\hat{\mu }}_{k}^{-}\right)\right)\right]}_{}^{\vee }={\left(\tau_{lra}^{}\left({\hat{\mu }}_{k}^{-}\right)\right)}^{T}\tau_{lra}^{}\left({\hat{\mu }}_{k}^{-}\right) \end{array}$$

Then solve for $\tau_{lra}^{}\left({\hat{\mu }}_{k}^{-}\exp\left({\left[\epsilon \right]}_{G}^{\wedge }\right)\right)$ and ${\left[\log\left(h_{lra}^{}\left({\hat{\mu }}_{k}^{-}\exp\left({\left[\epsilon \right]}_{G}^{\wedge }\right)\right)\right)\right]}_{}^{\vee }$ as shown in Equations (42) and (43).

(42) $$\begin{array}{c} \begin{array}{cc} \tau_{lra}^{}\left({\hat{\mu }}_{k}^{-}\exp\left({\left[\epsilon \right]}_{G}^{\wedge }\right)\right) & =E({\hat{T}}_{k}^{ls-}\exp\left({\left[\epsilon_{T}^{ls}\right]}_{}^{\wedge }\right){pls}_{}^{la}-{\hat{T}}_{k}^{rs-}\exp\left({\left[\epsilon_{T}^{rs}\right]}_{}^{\wedge }\right){prs}_{}^{ra})\\ & \mathrm{Take}\;\mathrm{the}\;1\mathrm{st}\;\mathrm{order}\;\mathrm{approximation}\\ & \approx E({\hat{T}}_{k}^{ls-}\;{pls}_{}^{la}-{\hat{T}}_{k}^{rs-}\;{prs}_{}^{ra}+{\hat{T}}_{k}^{ls-}{\left[\epsilon_{T}^{ls}\right]}_{}^{\wedge }{pls}_{}^{la}-{\hat{T}}_{k}^{rs-}{\left[\epsilon_{T}^{rs}\right]}_{}^{\wedge }{prs}_{}^{ra})\\ & =\tau_{lra}^{}\left({\hat{\mu }}_{k}^{-}\right)+\overset{\Gamma_{lra}}{\overset{︷}{E\left({\hat{T}}_{k}^{ls-}{\left[{pls}_{}^{la}\right]}_{}^{⊙}\epsilon_{T}^{ls}-{\hat{T}}_{k}^{rs-}{\left[{prs}_{}^{ra}\right]}_{}^{⊙}\epsilon_{T}^{rs}\right)}},\;\mathrm{Using}\;\mathrm{Equation}\;(11) \end{array} \end{array}$$

(43)
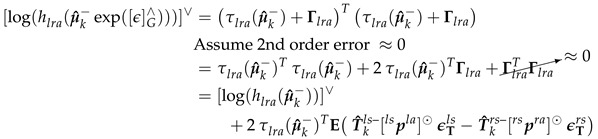

(44)
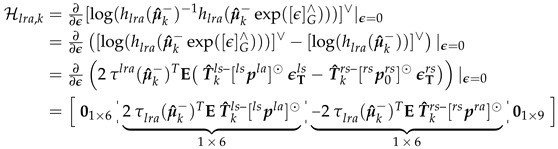


##### Pelvis-to-Ankle Distance Measurement

In addition to the soft ankle distance constraint, the ankle to pelvis vector is inferred from the ankle to pelvis distance measurements while assuming hinged knee joints and constant body segment lengths. The measurement function is defined by Equation (45), with measurement noise variance $\sigma_{pla}^{2}$ (3×1 vector), where ${pp}_{}^{mp}$ is the position of the mid-pelvis expressed in pelvis frame, and ${pls}_{}^{la}$ is the position of the left ankle expressed in left shank frame. We have chosen that the mid-pelvis and ankle are at the origin of their corresponding reference frames.

(45) $$\begin{array}{c} {\left[\log\left(h_{pla}^{}\left(X_{k}\right)\right)\right]}_{}^{\vee }=E\left(\overset{\mathrm{mid}-\mathrm{pelvis}\;\mathrm{in}\;W}{\overset{︷}{T_{k}^{p}\;{pp}_{}^{mp}}}-\overset{\mathrm{left}\;\mathrm{ankle}\;\mathrm{in}\;W}{\overset{︷}{T_{k}^{ls}\;{pls}_{}^{la}}}\right)\in R^{3},\;{pp}_{}^{mp}={pls}_{}^{la}={\left[\begin{array}{cccc} 0 & 0 & 0 & 1 \end{array}\right]}^{T} \end{array}$$

The measurement pelvis to left ankle vector can be calculated from the measured pelvis to left ankle distance, ${\overset{˘}{d}}_{k}^{pla}$ as shown in Equation (46) which is the Lie Group reformulation of [31] (Equation (4)). In essence, Equation (47) calculates the most probably knee angle assuming hinged knee joint and constant body segment lengths, then Equation (46) adds the thigh (expressed in shank coordinate system with knee angle ${\hat{\theta }}_{k}^{lk}$) and shank long axis to the hips to obtain the pelvis-to-ankle vector. See Appendix A for derivation. There are two solutions for ${\hat{\theta }}_{k}^{lk}$ due to the inverse cosine in Equation (47). We chose the ${\hat{\theta }}_{k}^{lk}$ value as that closer to the current left knee angle estimate from the prediction step. Note that this measurement function could also be formulated as a linearized Euclidean distance between the pelvis and ankle (i.e., similar to Equation (44)); however, a preliminary exploration of this approach showed poorer performance.

(46) $$\begin{array}{c} {\left[\log\left(Z_{pla,k}^{}\right)\right]}_{}^{\vee }=\overset{\psi_{pla}=\mathrm{half}\;\mathrm{pelvis}\;y-\mathrm{axis}\;+\;\mathrm{shank}\;z-\mathrm{axis}}{\overset{︷}{\frac{d^{p}}{2}{\hat{T}}_{k}^{p-}\;i_{y}^{}-d^{ls}{\hat{T}}_{k}^{ls-}\;i_{z}^{}}}+d^{lt}{\hat{T}}_{k}^{ls-}\overset{\mathrm{thigh}\;z-\mathrm{axis}\;\mathrm{in}\;\mathrm{shank}\;\mathrm{frame}}{\overset{︷}{(i_{x}^{}\sin\left({\hat{\theta }}_{k}^{lk}\right)-i_{z}^{}\cos\left({\hat{\theta }}_{k}^{lk})\right)}}\in R^{3} \end{array}$$

(47) $$\begin{array}{c} {\hat{\theta }}_{k}^{lk}=\cos^{-1}\left(\frac{\alpha \gamma \pm \beta \sqrt{\alpha^{2}+\beta^{2}-\gamma^{2}}}{\alpha^{2}+\beta^{2}}\right)\;\mathrm{where}\;\;\begin{array}{cc} & \alpha =-2d^{lt}\psi_{pla}^{T}{\hat{T}}_{k}^{ls-}\;i_{z}^{},\;\beta =2d^{lt}\psi_{pla}^{T}{\hat{T}}_{k}^{ls-}\;i_{x}^{},\;\\ & \gamma ={\left({\overset{˘}{d}}_{k}^{pla}\right)}^{2}-\psi_{pla}^{T}\psi_{pla}-{\left(d^{lt}\right)}^{2} \end{array} \end{array}$$

To calculate for $H_{pla,k}$, we first solved for ${\left[\log\left(h_{pla}^{}\left(X_{k}\right)\right)\right]}_{}^{\vee }$ at $X_{k}={\hat{\mu }}_{k}^{-}$ similar to Equation (41).

(48) $$\begin{array}{c} {\left[\log\left(h_{pla}^{}\left({\hat{\mu }}_{k}^{-}\right)\right)\right]}_{}^{\vee }=\tau_{pla}^{}\left({\hat{\mu }}_{k}^{-}\right)=E\left({\hat{T}}_{k}^{p-}{pp}_{}^{mp}-{\hat{T}}_{k}^{ls-}{pls}_{}^{la}\right) \end{array}$$

Then solve for ${\left[\log\left(h_{pla}^{}\left({\hat{\mu }}_{k}^{-}\exp\left({\left[\epsilon \right]}_{G}^{\wedge }\right)\right)\right)\right]}_{}^{\vee }$ similar to Equation (42) (i.e., distance between mid-pelvis and left ankle) giving us ${\left[\log\left(h_{pla}^{}\left({\hat{\mu }}_{k}^{-}\exp\left({\left[\epsilon \right]}_{G}^{\wedge }\right)\right)\right)\right]}_{}^{\vee }=\tau_{pla}^{}\left({\hat{\mu }}_{k}^{-}\right)+\Gamma_{pla}$. $H_{pla,k}$ is then calculated as shown in Equation (49). The right side of the pelvis-to-ankle distance measurement (i.e., $h_{pra}\left({\hat{\mu }}_{k}^{-}\right)$, $Z_{pra}$, $H_{pra,k}$) can be solved similarly to the left side.
(49)
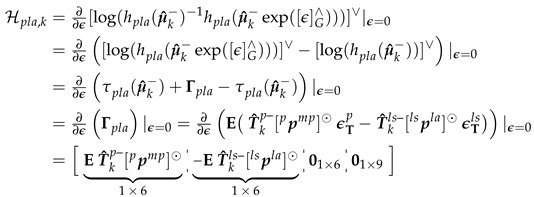


##### Covariance Limiter

Lastly, the error covariance of the position estimates of the three instrumented body segments must be prevented from growing unbounded and/or becoming badly conditioned, as will occur naturally when tracking global position of objects without any global position reference. At this step, a pseudo-measurement equal to the current state ${\hat{\mu }}_{k}^{+}$ is used (implemented by Equation (50)) with some measurement noise of variance $\sigma_{lim}^{}$ (9×1 vector). The covariance $P_{k}^{+}$ is then calculated through Equations (51)–(53).
(50)
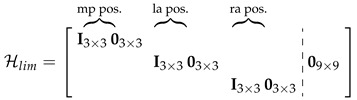

(51) $$\begin{array}{c} H_{k}^{\prime }={\left[H_{k}^{T}\;H_{lim}^{T}\right]}^{T},\;R_{k}^{\prime }=\mathrm{diag}\left(\sigma_{k}^{2},\sigma_{lim}^{2}\right) \end{array}$$

(52) $$\begin{array}{c} K_{k}^{\prime }=P_{k}^{-}H_{k}^{\prime T}{\left(H_{k}^{\prime }P_{k}^{-}H_{k}^{\prime T}+R^{\prime }\right)}^{-1} \end{array}$$

(53) $$\begin{array}{c} P_{k}^{+}=\Phi_{G}\left(\nu_{k}\right)\left(I-K_{k}^{\prime }H_{k}^{\prime }\right)P_{k}^{-}\Phi_{G}{\left(\nu_{k}\right)}^{T} \end{array}$$

#### 3.2.3. Satisfying Biomechanical Constraints

After the preceding updates, the joint positions or angles may be beyond their allowed range (i.e., knee hyperflexion). The constraint update corrects the kinematic state estimates to satisfy the biomechanical constraints of the human body by projecting the current a posteriori state estimate ${\hat{\mu }}_{k}^{+}$ onto the constraint surface, guided by our uncertainty in each state variable, which is encoded by $P_{k}^{+}$. The following biomechanical constraint equations are enforced: (i) estimated thigh long axis vector lengths equal the thigh lengths; (ii) both knees act as hinge joints (formulation similar to Section 2.3, Equation (4) of [9]); and (iii) the knee joint angle is within realistic range. The constraint functions are similar to Section II-E.3 of [15] but expressed under SE(3) state variables. The constrained state ${\tilde{\mu }}_{k}^{+}$ can be calculated using the equations below, similar to the measurement update of [36] with zero noise, where $C_{k}={\left[C_{L,k}^{T}\;C_{R,k}^{T}\right]}^{T}$. $C_{L,k}$ is the concatenation of $C_{ltl,k}$, $C_{lkh,k}$, and $C_{lkr,k}$; the last matrix is not concatenated when the knee angle, $\alpha_{lk}$, is within its allowed range (i.e., $\alpha_{lk,min}\leq \alpha_{lk}\leq \alpha_{lk,max}$). $C_{ltl,k}$, $C_{lkh,k}$, and $C_{lkr,k}$ corresponds to the biomechanical constraint for the left thigh length (*ltl*), left knee hinged joint (*lkh*), and left knee angle ROM (*lkr*), respectively, which will be described more later. $C_{R,k}$ can be derived similarly, while $D_{k}$ and $c({\hat{\mu }}_{k}^{+}))$ are constructed similarly to $Z_{k}$.

(54) $$\begin{array}{c} {\tilde{\mu }}_{k}^{+}={\hat{\mu }}_{k}^{+}\exp\left({\left[\nu_{k}\right]}_{G}^{\wedge }\right) \end{array}$$

(55) $$\begin{array}{c} \nu_{k}=K_{k}({\left[\log\left(c{\left({\hat{\mu }}_{k}^{+}\right)}^{-1}D_{k}\right)\right]}_{G_{c}}^{\vee } \end{array}$$

(56) $$\begin{array}{c} K_{k}=P_{k}^{+}C_{k}^{T}{\left(C_{k}P_{k}^{+}C_{k}^{T}\right)}^{-1}) \end{array}$$

(57) $$\begin{array}{c} C_{k}=\frac{\partial }{\partial \epsilon }{\left[\log\left(c{\left({\hat{\mu }}_{k}^{+}\right)}^{-1}c\left({\hat{\mu }}_{k}^{+}\exp\left({\left[\epsilon \right]}_{G}^{\wedge }\right)\right)\right)\right]}_{G_{c}}^{\vee }{|}_{\epsilon =0} \end{array}$$

##### Thigh Length Constraint

Firstly, the thigh length constraint is shown in Equation (59), where $\tau_{z}^{lt}\left(X_{k}\right)$ (Equation (58)) denotes the thigh long axis vector and $d^{lt}$ denotes the measured thigh length during calibration. ${pp}_{}^{lh}$ is the position of the left hip expressed in pelvis frame, and ${pls}_{}^{lk}$ is the position of the left knee expressed in left shank frame. We have chosen that the left hip to be $\frac{d_{}^{p}}{2}$ to the left of the mid-pelvis origin, and the left knee to be $d_{}^{ls}$ from the left shank origin (i.e., from the left ankle).

(58) $$\begin{array}{c} \tau_{z}^{lt}\left(X_{k}\right)=E(\overset{\mathrm{hip}\;\mathrm{jt}.\;\mathrm{pos}.\;\mathrm{in}\;W}{\overset{︷}{T_{}^{p}\;{pp}_{}^{lh}}}-\overset{\mathrm{knee}\;\mathrm{jt}.\;\mathrm{pos}.\;\mathrm{in}\;W}{\overset{︷}{T_{}^{ls}\;{pls}_{}^{lk}}}),\;{pp}_{}^{lh}={\left[\begin{array}{cccc} 0 & \frac{d^{p}}{2} & 0 & 1 \end{array}\right]}^{T},\;{pls}_{}^{lk}={\left[\begin{array}{cccc} 0 & 0 & d^{ls} & 1 \end{array}\right]}^{T} \end{array}$$

(59) $$\begin{array}{c} \log\left(c_{ltl}^{}\left(X_{k}\right)\right){]}_{}^{\vee }={\left(\tau_{z}^{lt}\left(X_{k}\right)\right)}^{T}\tau_{z}^{lt}\left(X_{k}\right)\in R,\;{\left[\log\left(D_{ltl}^{}\right)\right]}_{}^{\vee }={\left(d^{lt}\right)}^{2} \end{array}$$

$C_{ltl,k}$ is calculated using Equation (60).

(60) $$\begin{array}{c} \begin{array}{cc} C_{ltl,k} & =\frac{\partial }{\partial \epsilon }{\left[\log\left(c_{ltl}^{}{\left({\hat{\mu }}_{k}^{+}\right)}^{-1}c_{ltl}^{}\left({\hat{\mu }}_{k}^{+}\exp\left({\left[\epsilon \right]}_{G}^{\wedge }\right)\right)\right)\right]}_{}^{\vee }{|}_{\epsilon =0}\\ & =\frac{\partial }{\partial \epsilon }\left({\left[\log\left(c_{ltl}^{}\left({\hat{\mu }}_{k}^{+}\exp\left({\left[\epsilon \right]}_{G}^{\wedge }\right)\right)\right)\right]}_{}^{\vee }-{\left[\log\left(c_{ltl}^{}\left({\hat{\mu }}_{k}^{+}\right)\right)\right]}_{}^{\vee }\right){|}_{\epsilon =0} \end{array} \end{array}$$

Following similar procedure to $H_{lra,k}$, we obtain $\tau_{z}^{lt}\left({\hat{\mu }}_{k}^{+}\exp\left({\left[\epsilon \right]}_{G}^{\wedge }\right)\right)=\tau_{z}^{lt}\left({\hat{\mu }}_{k}^{+}\right)+\Gamma_{ltz}$ (similar to Equation (42)), and ${\left[\log\left(c_{ltl}^{}\left({\hat{\mu }}_{k}^{+}\exp\left({\left[\epsilon \right]}_{G}^{\wedge }\right)\right)\right)\right]}_{}^{\vee }={\left[\log\left(c_{ltl}^{}\left({\hat{\mu }}_{k}^{+}\right)\right)\right]}_{}^{\vee }+2{\left(\tau_{z}^{lt}\left({\hat{\mu }}_{k}^{+}\right)\right)}^{T}E({\hat{T}}_{k}^{p+}{\left[{pp}_{}^{lh}\right]}_{}^{⊙}\epsilon_{T}^{p}-{\hat{T}}_{k}^{ls+}{\left[{pls}_{}^{lk}\right]}_{}^{⊙}\epsilon_{T}^{ls})$ (similar to Equation (43)), which if we substitute in Equation (60) gives us Equation (61)
(61)
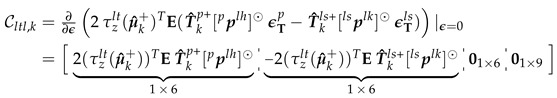


##### Hinge Knee Joint Constraint

Secondly, the hinge knee joint constraint as defined by Equation (62) is enforced by having the long (*z*) axis of the thigh to be perpendicular to the mediolateral axis (*y*) of the shank. For example, on the left leg, we would want $r_{y}^{ls}$ be perpendicular to the thigh long axis vector $\tau_{z}^{lt}\left({\hat{\mu }}_{k}^{+}\right)$ (i.e., the dot product of $r_{y}^{ls}$ and $\tau_{z}^{lt}\left({\hat{\mu }}_{k}^{+}\right)$ should be 0). Refer to Figure 2 for visualization. This formulation is similar to Section 2.3, Equation (4) of [9].

(62) $$\begin{array}{c} {\left[\log\left(c_{lkh}^{}\left(X_{k}\right)\right)\right]}_{}^{\vee }={\left(ET_{}^{ls}i_{y}\right)}^{T}\tau_{z}^{lt}\left(X_{k}\right)={\left(r_{y}^{ls}\right)}^{T}\tau_{z}^{lt}\left(X_{k}\right)\in R,\;{\left[\log\left(D_{lkh}^{}\right)\right]}_{}^{\vee }=0 \end{array}$$

Following similar procedure to $C_{ltl,k}$ and taking $X_{k}={\hat{\mu }}_{k}^{+}$, ${\left[\log\left(c_{lkh}^{}\left({\hat{\mu }}_{k}^{+}\right)\right)\right]}_{}^{\vee }$ and ${\left[\log\left(c_{lkh}^{}\left({\hat{\mu }}_{k}^{+}\exp\left({\left[\epsilon \right]}_{G}^{\wedge }\right)\right)\right)\right]}_{}^{\vee }$ can be calculated as shown in Equations (63) and (64), respectively.

(63) $$\begin{array}{c} {\left[\log\left(c_{lkh}^{}\left({\hat{\mu }}_{k}^{+}\right)\right)\right]}_{}^{\vee }={\left(E{\hat{T}}_{}^{ls+}i_{y}\right)}^{T}\tau_{z}^{lt}\left({\hat{\mu }}_{k}^{+}\right) \end{array}$$

(64)
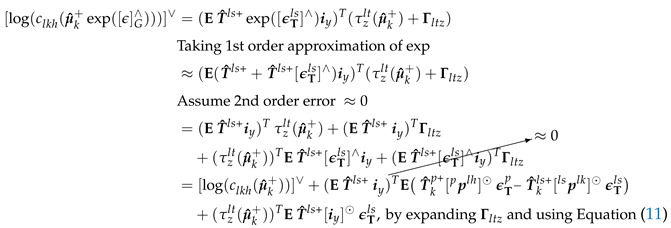


$C_{lkh,k}$ can be calculated using Equation (65).

(65) $$\begin{array}{c} \begin{array}{cc} C_{lkh,k} & =\frac{\partial }{\partial \epsilon }{\left[\log\left(c_{lkh}^{}{\left({\hat{\mu }}_{k}^{+}\right)}^{-1}c_{lkh}^{}\left({\hat{\mu }}_{k}^{+}\exp\left({\left[\epsilon \right]}_{G}^{\wedge }\right)\right)\right)\right]}_{}^{\vee }{|}_{\epsilon =0}\\ & =\frac{\partial }{\partial \epsilon }\left({\left[\log\left(c_{lkh}^{}\left({\hat{\mu }}_{k}^{+}\exp\left({\left[\epsilon \right]}_{G}^{\wedge }\right)\right)\right)\right]}_{}^{\vee }-{\left[\log\left(c_{lkh}^{}\left({\hat{\mu }}_{k}^{+}\right)\right)\right]}_{}^{\vee }\right){|}_{\epsilon =0} \end{array} \end{array}$$

Substituting Equations (63) and (64) into Equation (65) gives us Equation (66).
(66)
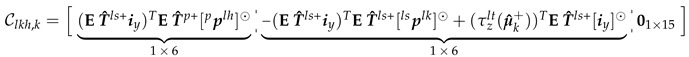


##### Knee Range of Motion Constraint

Thirdly, the knee ROM constraint is defined by Equation (69) and is only enforced if the knee angle, $\alpha_{lk}$, is outside the allowed ROM. The bounded knee angle, $\alpha_{lk}^{\prime }$, is calculated by Equation (67). Equation (69) is obtained by expanding Equation (67) to Equation (68) which when rearranged gives us ${\left[\log\left(c_{lkr}^{}\left(X_{k}\right)\right)\right]}_{}^{\vee }$ (i.e., Lie group representation of Equation (26) in [15]). Note that ${rls}_{z}^{lt}$ is the normalized thigh long axis expressed in the left shank frame.

(67) $$\begin{array}{c} \alpha_{lk}=\tan^{-1}\left(\frac{-{\left(r_{z}^{ls}\right)}^{T}r_{z}^{lt}}{-{\left(r_{x}^{ls}\right)}^{T}r_{z}^{lt}}\right)+\frac{\pi }{2},\;\alpha_{lk}^{\prime }=\min\left(\alpha_{lk,max},\max\left(\alpha_{lk,min},\alpha_{lk}\right)\right) \end{array}$$

(68) $$\begin{array}{c} \frac{-r_{z}^{lt}\cdot r_{z}^{ls}}{-r_{z}^{lt}\cdot r_{x}^{ls}}=\frac{\sin(\alpha_{lk}^{\prime }-\frac{\pi }{2})}{\cos(\alpha_{lk}^{\prime }-\frac{\pi }{2})} \end{array}$$

(69) $$\begin{array}{c} {}[\log\left(c_{lkr}^{}\left(X_{k}\right)\right){]}_{}^{\vee }={\left(E\;T_{}^{ls}\overset{{rls}_{z}^{lt}=\mathrm{long}\;\mathrm{axis}\;\mathrm{of}\;\mathrm{left}\;\mathrm{thigh}\;\mathrm{in}\;\mathrm{shank}\;\mathrm{frame}}{\overset{︷}{\left(i_{z}\cos\left(\alpha_{lk}^{\prime }-\frac{\pi }{2}\right)-i_{x}\sin\left(\alpha_{lk}^{\prime }-\frac{\pi }{2}\right)\right)}}\right)}^{T}\tau_{z}^{lt}\left(X_{k}\right)\in R,\;{\left[\log\left(D_{lkr}^{}\right)\right]}_{}^{\vee }=0 \end{array}$$

Following a similar procedure to $C_{lkh,k}$ (i.e., replace $i_{y}^{}$ in Equation (64) with ${rls}_{z}^{lt}$) and taking $X_{k}={\hat{\mu }}_{k}^{+}$, $C_{lkr,k}$ can be calculated from $c_{lkr}\left({\hat{\mu }}_{k}^{+}\exp\left({\left[\epsilon \right]}_{G}^{\wedge }\right)\right)={\left[\log\left(c_{lkr}^{}\left({\hat{\mu }}_{k}^{+}\right)\right)\right]}_{}^{\vee }+{\left(E\;{\hat{T}}_{}^{ls+}{rls}_{z}^{lt}\right)}^{T}E\left({\hat{T}}_{k}^{p+}{\left[{pp}_{}^{lh}\right]}_{}^{⊙}\epsilon_{T}^{p}-{\hat{T}}_{k}^{ls+}{\left[{pls}_{}^{lk}\right]}_{}^{⊙}\epsilon_{T}^{ls}\right)+{\left(\tau_{z}^{lt}\left({\hat{\mu }}_{k}^{+}\right)\right)}^{T}E\;{\hat{T}}_{}^{ls+}{\left[{rls}_{z}^{lt}\right]}_{}^{⊙}\epsilon_{T}^{ls}$, as shown in Equation (70).
(70)
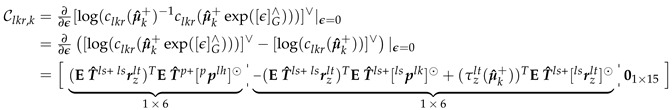


### 3.3. Post-Processing

The orientation of the pelvis and shanks are obtained from the state ${\tilde{\mu }}_{k}^{+}$. The orientation of the left thigh, ${\tilde{R}}_{}^{lt+}$, can be calculated using ${\tilde{R}}_{}^{lt+}$ = 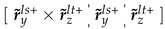 = 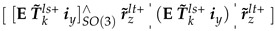, where ${\tilde{r}}_{z}^{lt+}=\tau_{z}^{lt}\left({\tilde{\mu }}_{k}^{+}\right)\;/\;\left|\right|\tau_{z}^{lt}\left({\tilde{\mu }}_{k}^{+}\right)\left|\right|$. The orientation of the right thigh, ${\tilde{R}}_{}^{rt+}$, is calculated similarly.

## 4. Experiment

An extension of the dataset from [15] was used to evaluate our L5S based algorithms. The movements involved are listed in Table 1 (note the addition of dynamic movements), and were collected from from nine healthy subjects (7 men and 2 women, weight 63.0±6.8 kg, height 1.70±0.06 m, age 24.6±3.9 years old), with no known gait abnormalities. Raw data were captured using a commercial IMC (i.e., Xsens Awinda) at 100 Hz sampling rate with IMUs attached to the pelvis and ankles, compared against a benchmark OMC (i.e., the setup followed Vicon Plug-in Gait protocol in a ∼4×4 m${}^{2}$ capture area). The experiment was approved by the Human Research Ethics Board of the University of New South Wales (UNSW) with approval number HC180413.

Frame alignment and yaw offset calibrations are similar to Section III-B of [15]. The experiments and algorithm were implemented using Matlab 2020a, with initial state ${\tilde{\mu }}_{0}^{+}$ (i.e., position, orientation, and velocity) obtained from the OMC system (i.e., Vicon) and initial error covariance $P_{0}^{+}$ set to $0.5I_{27\times 27}$. The variance parameters for the process and measurement error covariance matrix Q and R are shown in Table 2.

The inter-IMU distance measurements, ${\overset{˘}{d}}^{pla}$, ${\overset{˘}{d}}^{pra}$, and ${\overset{˘}{d}}^{lra}$, were simulated by calculating the distance from the mid-pelvis to the left and right ankles and adding normally distributed positional noise with different standard deviations (i.e., $\sigma_{dist}\in \left\{0,0.01,\cdots ,0.1,0.15,0.2\right\}$ m). Each trial was simulated five times.

Lastly, the evaluation was done using the following metrics: (1) Mean position and orientation root-mean-square error (RMSE) (e.g., similar to [15,17] as shown in Equations (71) and (72)), where $p_{k}^{b}$ and $R_{k}^{b}$ are obtained from the benchmark OMC system, ${\tilde{p}}_{k}^{b+}$ and ${\tilde{R}}_{k}^{b+}$ are obtained from the algorithm. Note that as the global position of the estimate is still prone to drift due to the absence of an external global position reference, the root position of our system was set equal to that of the benchmark system (i.e., the mid-pelvis is placed at the origin in the world frame for all RMSE calculations). (2) Joint angles RMSE with bias removed (i.e., the mean difference between the angles over each entire trial was subtracted) and correlation coefficient (CC) of the hip in the sagittal (Y), frontal (X), and transverse (Z) planes and of the knee in the sagittal (Y) plane. Note that these joint angles are commonly used parameters in gait analysis. (3) Spatiotemporal gait parameters (e.g., total travelled distance (TTD) deviation, average stride length, and gait speed of the foot). Refer to Section III of [15] for more details.

(71) $$\begin{array}{c} e_{pos,k}=\frac{1}{N_{pos}}\sum_{b\in \mathrm{DP}}\left|\right|p_{k}^{b}-{\tilde{p}}_{k}^{b+}\left|\right|,\;N_{pos}=6,\;\mathrm{DP}=\left\{lh,rh,lk,rk,la,ra\right\} \end{array}$$

(72) $$\begin{array}{c} e_{ori,k}=\frac{1}{N_{ori}}\sum_{b\in \mathrm{DO}}\left|\right|{\left[\log\left(R_{k}^{b}{\left({\tilde{R}}_{k}^{b+}\right)}^{T}\right)\right]}_{}^{\vee }\left|\right|,\;N_{ori}=2,\;\mathrm{DO}=\left\{lt,rt\right\} \end{array}$$

## 5. Results

### 5.1. Mean Position and Orientation RMSE, Joint Angle RMSE and CC

In this experiment, multiple variations of the algorithm were tested as shown in Table 3. Firstly, L5S-3IMU is the algorithm described in this paper (Section 3) with parameters listed in Table 2. The parameter for L5S-3IMU were selected by taking the best joint CC (i.e., mean of free walk and dynamic movements) from a grid search of parameters $\sigma_{\omega }^{2}=\left\{1,10,10^{2},10^{3}\right\}$ rad${}^{2}$/s${}^{2}$ and $\sigma_{ori}^{2}=\left\{10^{-2},10^{-1},1,10\right\}$ rad${}^{2}$. Secondly, CKF-3IMU and CKF-3IMU+D were the algorithms described in [15,31], respectively. Thirdly, CKF-3I-KB is a modified CKF-3IMU using similar parameters, measurement, and constraint functions as L5S-3IMU. The key difference between CKF-3IMU and CKF-3I-KB is that CKF-3I-KB allows knee bending, denoted by the suffix KB, during the constraint update. Fourthly, L5S-3I-NO is a variation of L5S-3IMU with $\sigma_{\omega }^{2}=10^{7}$ rad${}^{2}$/s${}^{2}$, $\sigma_{ori}^{2}=10^{-1}$ rad${}^{2}$, and ${\overset{˘}{\omega }b}_{k}^{}=0$ rad. The parameters were chosen to have high uncertainty on the tracked orientation (i.e., effectively not using the orientation measurements at all), leading to a variation of L5S-3IMU that is similar to our prior work CKF-3IMU which assumed orientation measurements were noise-free. Lastly, the black box output (i.e., pelvis, thigh, and shank orientations) from the MVN Studio software (denoted as OSPS), which illustrates the performance of a widely-accepted commercial wearable IMC system with an OSPS configuration. For the first to fourth variations, the +D suffix means simulated inter-IMU distance measurements ($\sigma_{dist}=0.1$ m) was used instead of the pelvis height assumption.

**Figure 4:**
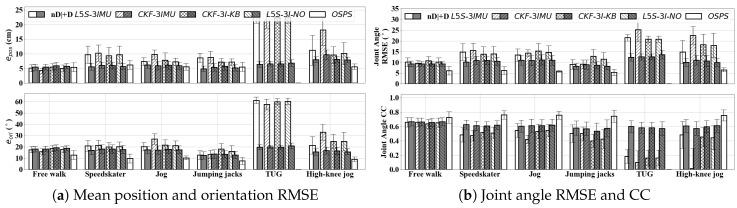
The performance of CKF, L5S, and OSPS with and without using inter-IMU distance measurements at each motion type.

Figure 4 shows the mean position and orientation RMSE, mean knee Y and hip joint angle RMSE (bias removed) and CC of different variations of CKF-3IMU and L5S-3IMU for both free walking and dynamic motions. Y, X, and Z refers to the sagittal, frontal, and transverse planes, respectively. CKF-3IMU performed well with free walking ($e_{pos}=4.27$ cm, $e_{ori}=15.85^{\circ }$, CC=0.66) [15]. However, a more extensive evaluation showed that it performed poorly for certain dynamic movements (e.g., high-knee jog with $e_{pos}=18.15$ cm, $e_{ori}=24.87^{\circ }$, CC=0.02). Removing the no-knee-bending assumption during the constraint update fixed this issue, as shown by the performance of CKF-3I-KB (e.g., high-knee jog improved by ∼9 cm $e_{pos}$, ∼$9^{\circ }$ $e_{ori}$, ∼0.4 CC). L5S-3I-NO which is the L5S version of CKF-3IMU expectedly have similar performance with CKF-3I-KB (i.e., $\Delta e_{pos}<0.5$ cm, $\Delta e_{ori}<1^{\circ }$, and ΔCC 0.02 differences). L5S-3IMU, which tracked both position and orientation while assuming there is noise in the orientation measurements, had a slightly better performance (e.g., improved jumping jacks and high-knee jog by ∼0.1 CC, <0.03 CC difference with other movement types). The use of simulated distance measurement with $\sigma_{dist}=0.1$ m on CKF-3I-KB, L5S-3I-NO, and L5S-3IMU had slight effects for free walking, and a significant improvement for dynamic movements. For free walking, joint angle RMSE and CC of L5S-3IMU+D compared to L5S-3IMU improved by ∼$1^{\circ }$ and <0.01 CC, while $e_{pos}$ and $e_{ori}$ slightly disimproved (<0.5 cm and <1 ${}^{\circ }$). The similar results suggest that inferring pelvis position from simulated distance measurement ($\sigma_{dist}=0.1$ m) is comparable to our pelvis height assumption at least for free walking. For dynamic movements, the $e_{pos}$, $e_{ori}$, joint angle RMSE, and CC of L5S-3IMU+D improved by 2–16 cm, 0–40 ${}^{\circ }$, 1–9 ${}^{\circ }$, and <0.42, respectively—more significantly for movements TUG and high-knee jog.

**Figure 5:**
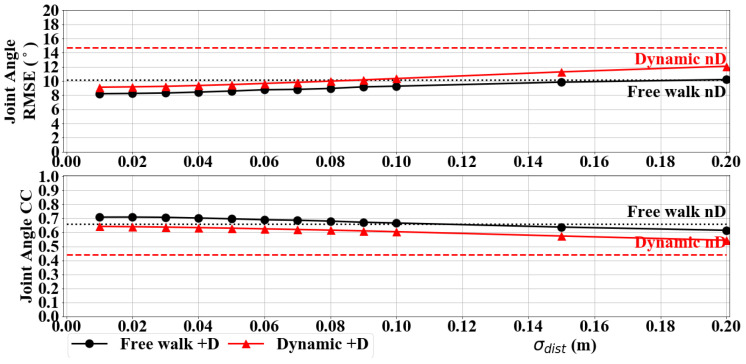
Joint angle RMSE (top) and CC (bottom) of free walk and dynamic movements at different noise level $\sigma_{dist}$. The broken lines represent L5S-3IMU results (denoted as nD) where inter-IMU distance measurements were not used. The solid lines represent L5S-3IMU+D results (denoted as +D) where we can observe slight and great improvements for free walk and dynamic movements, respectively.

To give insight on how the accuracy of the simulated inter-IMU distance measurements affect pose estimation performance, Figure 5 shows the mean of knee Y and hip joint angle RMSE and CC at different $\sigma_{dist}$ values. At $\sigma_{dist}=0.1$ m, the simulation showed comparable performance between L5S-3IMU, which implements pelvis height assumption, and L5S-3IMU+D, which implements inter-IMU distance measurement to supplement the pelvis position estimate, for free walking. Significant improvement for dynamic movements can be seen even for $\sigma_{dist}=0.2$ m. These results suggest that the actual distance measurement sensor must have noise standard deviation $\sigma_{dist}\leq 0.1$ m to improve pose estimate performance. Note that the +D variation in Figure 4 and in the experiments that follow were evaluated at $\sigma_{dist}=0.1$ m.

### 5.2. Hip and Knee Joint Angle RMSE and CC

**Figure 6:**
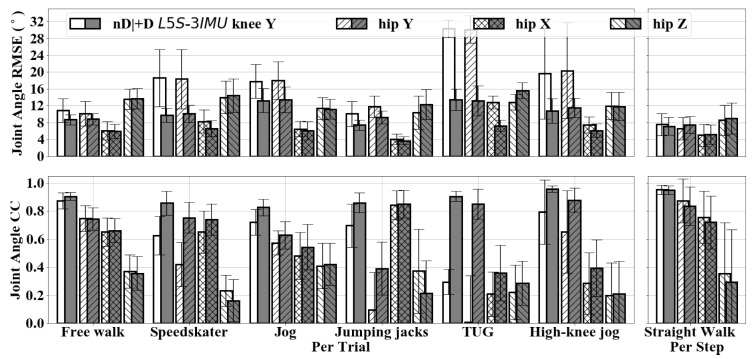
The CC of knee (Y) and hip (Y, X, Z) joint angles for L5S-3IMU (denoted as nD) and L5S-3IMU+D (denoted as +D) at each motion type.

Figure 6 shows the knee and hip joint angle RMSE (bias removed) and CC of L5S-3IMU and L5S-3IMU+D compared against the OMC output. Y, X, and Z refers to the sagittal, frontal, and transverse planes, respectively. Turning movements and half steps were manually removed from the per-step result of Walk movement and was denoted as Straight Walk. Note that sensor-to-body calibration was only done at the beginning of trial, not for each step. Between L5S-3IMU and L5S-3IMU+D, there was minimal hip and knee joint angle RMSE and CC improvement for free walking (∼$1^{\circ }$ RMSE and ∼0.03 CC difference). However, there was significant improvement for most dynamic movements, specifically, speed-skater, jog, high-knee jog, and TUG (e.g., $4^{\circ }$–$17^{\circ }$ knee Y and hip Y joint angle RMSE improvements). Furthermore, the CC for dynamic movements started to reach similar performance with the free walk movement, indicating that inter-IMU distance measurements have indeed made the pose estimator capable of tracking more ADLs and not just walking.

**Figure 7:**
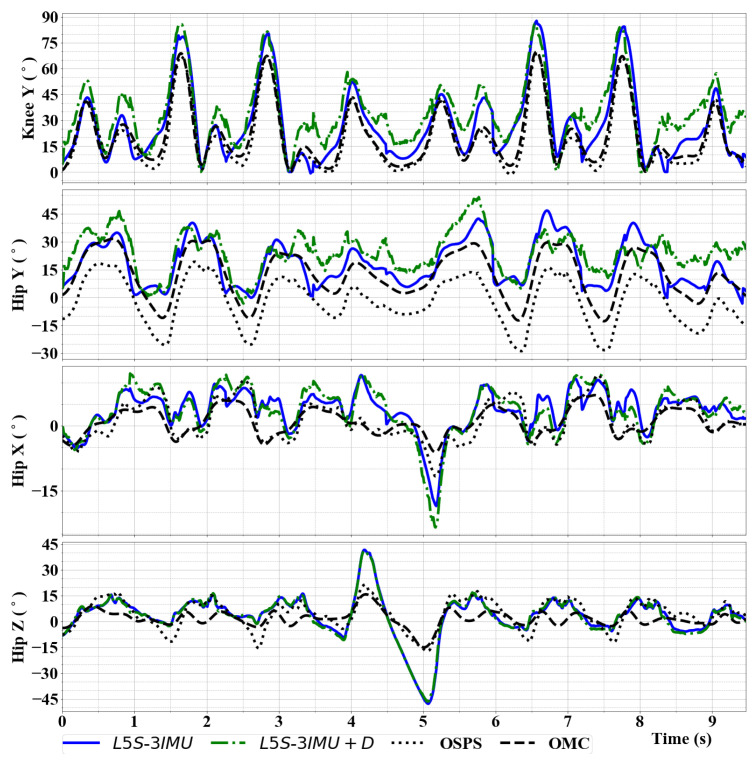
Knee (Y) and hip (Y, X, Z) joint angle output of L5S-3IMU in comparison with the benchmark system (Vicon) for a Walk trial. The subject walked straight from t=0 to 3 s, turned $180^{\circ }$ around from t=3 to 5.5 s, and walked straight to the original starting point from 5.5 s until the end.

Figure 7 shows a sample walk trial. At the peaks of knee Y angle, the distance between the pelvis and ankle positions of L5S-3IMU+D were a few cm shorter (i.e., pelvis position was lower than actual while ankle position was higher) than the actual distance resulting in higher knee Y angle peaks. Violations of our biomechanical constraints are also apparent at t=4 to 5.5 s, where the subject makes a $180^{\circ }$ turn. After the turn, L5S-3IMU and L5S-3IMU+D were able to recover during the straight walking (t=5.5 to 9.74 s of Figure 7). Notice that the bias between OSPS and OMC can be observed at t=0 of the hip Y joint angle.

### 5.3. Spatiotemporal Gait Parameters

Table 4 shows the TTD, stride length, and gait speed accuracy computed from the global ankle position estimate of L5S-3IMU, L5S-3IMU+D, and the OMC system for free walk, jogging, and TUG. The use of inter-IMU distance measurements ($\sigma_{dist}=0.1$ m) helped improve the TTD, stride length, and gait speed accuracy of free walk and TUG (e.g., TTD improved from ∼9% to ∼5%). Refer to the code repository for links to videos of sample trials.

## 6. Discussion

In this paper, a Lie group EKF algorithm for lower body pose estimation using only three IMUs, ergonomically placed on the ankles and sacrum to facilitate continuous recording outside the laboratory, was described and evaluated. The algorithm utilizes fewer sensors than other approaches reported in the literature, at the cost of reduced accuracy.

### 6.1. Mean Position and Orientation RMSE

The mean position and orientation RMSE of L5S-3IMU, L5S-3IMU+D, and related literature (sparse orientation poser (SOP) and sparse inertial poser (SIP) [17]) are listed in Table 5. SOP used orientation measured by IMUs and biomechanical constraints, while SIP used similar information but with the addition of acceleration. Both SOP and SIP were benchmarked against an OSPS system tracking the full body while our algorithm was benchmarked against an OMC system tracking only the lower body. The $e_{pos}$ and $e_{ori}$ (no bias) performance of L5S-3IMU and compared to SOP for free walking and jogging were comparable ($\Delta e_{pos}$ 0.1–0.5 cm and $\Delta e_{ori}$ 2.5${}^{\circ }$–3${}^{\circ }$ differences). The $e_{pos}$ and $e_{ori}$ (no bias) of SIP was better than L5S-3IMU and L5S-3IMU+D for free walking (∼2.1–2.5 cm and 6.5${}^{\circ }$–7${}^{\circ }$ difference). Although this improvement was expected, as SIP optimizes the pose over multiple frames whereas our algorithm, like CKF-3IMU, optimizes the pose for each individual frame. For jumping jacks, the $e_{ori}$ of L5S-3IMU and L5S-3IMU+D was significantly (∼4${}^{\circ }$–8${}^{\circ }$) better than SOP’s and SIP’s. However, this difference is probably because both SOP and SIP were evaluated on the full body (our algorithm was only evaluated on the lower body) and errors in arm pose estimation may have increased $e_{ori}$ for the SOP and SIP algorithms.

Comparing processing times, L5S-3IMU and L5S-3IMU+D were slower than CKF-3IMU, but can still be used in real-time; specifically, CKF-3IMU, L5S-3IMU, and L5S-3IMU+D processed a 1000-frame sequence (i.e., 10 s long) in ∼0.7, ∼2, ∼3.5 s, respectively, on an Intel Core i5-6500 3.2 GHz CPU [15], while SIP [17] took 7.5 min on a quad-core Intel Core i7 3.5 GHz CPU. All set-ups used single-core non-optimized Matlab code. Albeit slower than CKF-3IMU, L5S-3IMU and L5S-3IMU+D could also be used to provide real-time gait parameter measurement to inform actuation of assistive or rehabilitation robotic devices.

### 6.2. Hip and Knee Joint Angle RMSE and CC

The knee and hip joint angle RMSEs (no bias) and CCs of L5S-3IMU, L5S-3IMU+D, OSPS and related literature for straight walking (i.e., per step evaluation) are shown in Table 6 [7,15,37,38]. Similar to IMC based systems, L5S-3IMU and L5S-3IMU+D also follows the trend of having sagittal (Y axis) joint angles similar to that captured by OMC systems (0.95 knee Y and >0.83 hip Y CCs), but with significant difference in frontal and transverse (X and Z axis) joint angles [15,37]. CKF-3IMU performed slightly better (e.g., 0.03 knee Y, 0.09 hip Y CC), which is expected as the biomechanical constraint (i.e., no-knee-bending) assumption of CKF-3IMU was designed specifically for walking, at the cost of being less accurate for other dynamic movements. Both L5S-3IMU and L5S-3IMU+D were comparable, and at times even better (within $2.5^{\circ }$ RMSE, 0.1 CC difference) than the results of Hu et al. and Tadano et al., indicating excellent per-step reconstruction in the sagittal plane [7,38]. Hu et al. used 4 IMUs (two at the pelvis and one on each foot) and Tadano et al. used an OSPS configuration. Both systems can only estimate the pose in the sagittal plane.

Despite the promising performance when using inter-IMU distance measurements, further validation with actual hardware implementation is needed, as the sensor noise in the real world may not necessarily follow a normal distribution and may be non-stationary.

For reference, portable ultrasound-based distance measurement can achieve millimetre accuracy with a sampling rate of 125 Hz [30], while a commercial UWB-based distance measurement devices can achieve ∼10 cm accuracy with a sampling rate of 200 Hz [39,40].

Lastly, despite L5S-3IMU and L5S-3IMU+D achieving 0.95 joint angle CCs in the sagittal plane, the unbiased joint angle RMSE (>5${}^{\circ }$) makes its utility in clinical applications uncertain [41]. Although the algorithm is expected to work on pathological gait where our biomechanical assumptions are satisfied, overall performance still needs more improvement. To achieve clinical utility, one may either use more accurate sensors or average out cycle-to-cycle variation in estimation errors over many gait cycles; for example, use a more accurate distance measurement sensor ($\sigma_{dist}<0.1$ m). Furthermore, the accuracy must also be validated on a larger and more diverse cohort to quantify its ultimate clinical utility. The evaluation of how these solutions can bridge the gap to clinical application for the proposed system will be part of future work.

### 6.3. Spatiotemporal Gait Parameters

The focus of the proposed algorithms, L5S-3IMU and L5S-3IMU+D, are to estimate joint kinematics. However, as L5S-3IMU and L5S-3IMU+D both track the global position of the ankles, it is also capable of calculating spatiotemporal gait parameters (performance listed in Table 4). The TTD deviation of our algorithms compared against the gold standard OMC were not as good as CKF-3IMU [15] (3.6–3.81% TTD deviation) or other state-of-the-art dead reckoning algorithms [42,43] (0.2–1.5% TTD deviation). Two possible sources of inaccuracy lies (1) in the dead reckoning approximation done in the prediction step, and (2) in the assumption that the velocity of the shank IMU is zero when the associated foot touches the floor, but of course this IMU continues to move with some small velocity on the lower shank during the stance phase. To illustrate the dead reckoning approximation, let us look at the predicted pelvis pose in Equation (73). In our algorithm, we assumed $\psi_{p}\approx I_{3\times 3}$ (note that $\Phi_{}\left(-\Delta t{\overset{˘}{\omega }p}_{k}^{}\right)\approx I_{3\times 3}$ and ${\tilde{R}}_{k-1}^{p+}{\left({\overset{˘}{R}}_{k}^{p}\right)}^{T}\approx I_{3\times 3}$ since $\Delta t{\overset{˘}{\omega }p}_{k}^{}$ is small) which did not significantly affect the joint kinematic estimate, but slightly affected the global position estimate. Nevertheless, body drift has been reduced substantially compared to Marcard et al.’s SIP [17].

(73) $$\begin{array}{c} \begin{array}{cc} {\hat{T}}_{k}^{p-} & ={\tilde{T}}_{k-1}^{p+}\exp\left({\left[\left[\begin{array}{c} {\left({\overset{˘}{R}}_{k}^{p}\right)}^{T}\left(\Delta t{\tilde{v}}_{k-1}^{mp+}+\frac{\Delta t^{2}}{2}{\overset{˘}{a}}_{k}^{p}\right)\\ \Delta t{\overset{˘}{\omega }p}_{k}^{} \end{array}\right]\right]}_{}^{\wedge }\right)\\ & =\left[\begin{array}{cc} {\tilde{R}}_{k-1}^{p+}\exp\left({\left[\Delta t{\overset{˘}{\omega }p}_{k}^{}\right]}_{}^{\wedge }\right) & {\tilde{p}}_{k-1}^{mp+}+\overset{\psi_{p}\approx I_{3\times 3}}{\overset{︷}{\begin{array}{c} {\tilde{R}}_{k-1}^{p+}\Phi_{}\left(-\Delta t{\overset{˘}{\omega }p}_{k}^{}\right){\left({\overset{˘}{R}}_{k}^{p}\right)}^{T} \end{array}}}{\tilde{R}}_{k-1}^{p+}\Phi_{}\left(-\Delta t{\overset{˘}{\omega }p}_{k}^{}\right){\left({\overset{˘}{R}}_{k}^{p}\right)}^{T}\left(\Delta t{\tilde{v}}_{k-1}^{mp+}+\frac{\Delta t^{2}}{2}{\overset{˘}{a}}_{k}^{p}\right)\\ 0_{1\times 3} & 1 \end{array}\right] \end{array} \end{array}$$

### 6.4. Limitations and Future Work

L5S has similar pelvis drift, covariance matrix numerical issue, and flat floor limitation as CKF-3IMU, which is expected as L5S implements the same measurement and constraint update as CKF-3IMU, albeit formulated using Lie group representation instead of vectors and quaternions [15]. The pelvis height and flat floor assumption helps prevent the pelvis and the ankles from drifting towards each other (i.e., pelvis drift downward while ankles drift upward). However, it will also prevent accurate pose estimation of motions such as sitting, lying down, or standing on one leg, where the pose is maintained for a duration much longer than that of a typical gait cycle. The covariance limiter (Section 3.2.2) helps prevent the covariance becoming badly conditioned (i.e., singular), especially for longer duration trials (e.g., 5-minute walk) where the position uncertainty grows at a faster rate for the pelvis position than the ankle position. As can be observed from Figure 6, substituting the pelvis height assumption with inter-IMU distance measurements can increase the algorithm’s accuracy especially for tracking dynamic movements. If the distance measurement is accurate enough (i.e., smaller $\sigma_{dist}^{2}$), the inter-IMU distance measurement update may be enough to limit the growth of pelvis position uncertainly and possibly making the covariance limiter not needed.

Figure 6 shows that the optimized performance of L5S-3IMU, even if it allows the tracked orientation to be corrected by inter-IMU distance measurements and the tracked position estimate, was only slightly better than CKF-3IMU/L5S-3I-NO, which effectively assumed the measurement input from the orientation estimation algorithm to be perfect (i.e., trusted the tracked orientation less). As L5S-3IMU requires more computing resources, such result suggests that CKF-3IMU may be more suitable to use when computing power is limited. To fully leverage the advantages brought by the Lie group representation, additional sensor measurements that can help correct tracked orientation will be needed (e.g., estimating angle of arrival between two sensors [44] or using fish eye cameras to improve pose estimate [45]).

Additional sensor measurements provide new opportunities for automatic calibration even under RSC configuration. IMC systems typically need anthropometric measurements (i.e., measurement of body segments such as $d^{ls}$) beforehand. By taking the initial distance measurement at some predetermined posture, anthropometric measurements can be automatically inferred. The formulation for a hinge joint with two IMUs on both sides has been leveraged to enable automatic sensor-to-segment calibration (i.e., align sensor frame to body frame) and even a completely magnetometer free orientation estimation [46,47]. Magnetometer free orientation estimation rids us of the yaw offset issue from an inhomogeneous magnetic field in indoor environments, typically with stronger disturbances closer to the floor [48]. An approach using a hinge joint with two IMUs may not be applicable to RSC configurations (e.g., our algorithm only has one IMU on one side of the hinge joint). However, distance measurements may be use to compensate for the missing IMU information from the uninstrumented segment, and a modified version may be developed for a RSC configuration.

Enabling longer-term tracking of ADL in the subject’s natural environment may lead to novel investigations of movement disorder progression and the identification of early intervention opportunities. This work is just one of the early steps towards seamless remote gait monitoring. Developing solutions to further increase accuracy, increase the number of body segments tracked (e.g., track full body under RSC [17]), or use even fewer IMUs (tracking lower body using two IMUs [49]) will be investigated in the future.

## 7. Conclusions

This paper presented a Lie group CEKF-based algorithm (L5S-3IMU) to estimate lower limb kinematics using a RSC configuration of IMUs, supplemented by inter-IMU distance measurements in one implementation. The knee and hip joint angle RMSEs in the sagittal plane for straight walking were $7.6^{\circ }\pm 2.6^{\circ }$ and $6.6^{\circ }\pm 2.7^{\circ }$, respectively, while the CCs were 0.95±0.03 and 0.87±0.16, respectively. We also showed that inter-IMU distance measurement is a promising new source of information to improve the pose estimation of IMC under a RSC configuration. Simulations show that performance improved dramatically for dynamic movements even at higher noise levels (e.g., $\sigma_{dist}=0.2$ m), and that similar performance to L5S-3IMU was achieved at $\sigma_{dist}=0.1$ m for free walk movements. However, further validation is recommended with actual distance measurement from real sensors. The source code for the L5S algorithm, and links to sample videos will be made available at https://git.io/JTRQ3.

## Figures and Tables

**Table 1:** Types of movements done in the validation experiment.

Movement	Description	Duration	Group
Walk	Walk straight and return	∼30 s	F
Figure-of-eight	Walk along figure-of-eight path	∼60 s	F
Zig-zag	Walk along zig-zag path	∼60 s	F
5-minute walk	Unscripted walk and stand	∼300 s	F
Speedskater	Speedskater on the spot	∼30 s	D
TUG	Timed up-and-go test	∼30 s	D
Jog	Jog straight and return	∼30 s	D
Jumping jacks	Jumping jacks on the spot	∼30 s	D
High-knee jog	High-knee jog on the spot	∼30 s	D

F denotes free walk, D denotes dynamic movements.

**Table 2:** Parameters for error covariance matrices, Q and R.

Q Parameters		R Parameters					
$\sigma_{a}^{2}$	$\sigma_{\omega }^{2}$	$\sigma_{ori}^{2}$	$\sigma_{mp}^{2}$	$\sigma_{ls}^{2}$ and $\sigma_{rs}^{2}$	$\sigma_{dl}^{2}$ and $\sigma_{dr}^{2}$	$\sigma_{da}^{2}$	$\sigma_{lim}^{2}$
(m${}^{2}$·s${}^{-4}$)	(rad${}^{2}$·s${}^{-2}$)	(rad${}^{2}$)	(m${}^{2}$)	(m${}^{2}$·s${}^{-2}$ and m${}^{2}$)	(m${}^{2}$)	(m${}^{2}$)	(m${}^{2}$)
$10^{2}1_{9}$	$10^{3}1_{9}$	$11_{9}$	0.1	$\left[0.011_{3}\;10^{-4}\right]$	10	1	$101_{18}$

where $1_{n}$ is an 1×n row vector with all elements equal to 1.

**Table 3:** The experiment was tested on the following algorithm variations.

Algorithm	Inter-IMU Distance	Summary Description
L5S-3IMU	N	Tracks position and orientation as described in Section 3 with parameters listed in Table 2.
L5S-3IMU+D	Y	
CKF-3IMU [15]	N	Only tracks position using a constrained KF.
CKF-3IMU+D [31]	Y	
CKF-3I-KB	N	Modified CKF-3IMU using similar parameters as L5S-3IMU (Table 2). This also allows knee bending during the constraint update.
CKF-3I-KB+D	Y	
L5S-3I-NO	N	L5S-3IMU with parameters that assume noise-free orientation (NO) measurements like CKF-3IMU.
L5S-3I-NO+D	Y	
OSPS	N	Output from a commercial OSPS wearable IMC system.

**Table 4:** Total travelled distance (TTD) deviation from optical motion capture (OMC) system at the ankles.

Algo.	Side	TTD	Stride Length (cm)				Gait Speed (cm.s ${}^{-1}$)			
		Error	Actual		Error		Actual		Error	
		% dev	μ	med	μ ± σ	RMS	μ	med	μ ± σ	RMS
Freewalk	L	8.97%	91	99	−8.1±6	9.9	70	74	−6.0±5	7.7
L5S-3IMU	R	9.00%	93	99	−8.3±6	10.3	71	75	−6.2±5	8.2
Freewalk	L	5.23%	91	99	−4.7±7	8.3	70	74	−3.6±6	6.6
L5S-3IMU+D	R	5.85%	93	99	−5.4±8	9.4	71	75	−4.1±6	7.4
Jog	L	21.35%	81	86	−17.4±23	28.5	107	118	−19.2±33	38.0
L5S-3IMU	R	26.79%	85	97	−22.9±25	33.8	111	124	−26.4±34	43.1
Jog	L	22.40%	81	86	−18.2±22	28.4	107	118	−21.6±30	37.0
L5S-3IMU+D	R	26.70%	85	97	−22.8±24	32.8	111	124	−27.5±31	41.4
TUG	L	18.20%	74	76	−13.5±18	22.1	58	60	−10.0±15	18.0
L5S-3IMU	R	20.98%	79	90	−16.6±15	22.5	63	67	−13.1±13	18.4
ine TUG	L	3.80%	74	76	−2.8±6	6.7	58	60	−2.3±5	5.9
L5S-3IMU+D	R	4.22%	79	90	−3.3±6	6.8	63	67	−2.7±5	5.6

where μ and σ denote mean and standard deviation. Error denotes estimate minus actual value, while TTD % dev denotes abs(error)/actualTTD.

**Table 5:** Mean position and orientation RMSE of L5S-3IMU, L5S-3IMU+D, OSPS, sparse orientation power (SOP) and sparse inertial poser (SIP) [17].

	$e_{\mathrm{pos}}$ (cm)			$e_{\mathrm{ori}}$, No Bias (cm)		
	**Free Walk**	**Jog**	**Jumping Jacks**	**Free Walk**	**Jog**	**Jumping Jacks**
L5S-3I	5.1±1.2	7.3±1.4	8.7±1.6	$17.5\pm 2.7^{\circ }$	$20.2\pm 3.8^{\circ }$	$12.8\pm 4.0^{\circ }$
L5S-3I+D	5.5±1.0	6.2±1.1	4.9±0.9	$18.0\pm 2.5^{\circ }$	$17.4\pm 3.2^{\circ }$	$12.6\pm 3.2^{\circ }$
OSPS	5.4±1.5	5.6±1.2	5.5±1.6	$12.9\pm 4.0^{\circ }$	$10.3\pm 1.8^{\circ }$	$7.6\pm 3.3^{\circ }$
SOP [17]	∼5.0	∼8.0	∼8.0	∼$15.0^{\circ }$	∼$22.0^{\circ }$	∼$20.0^{\circ }$
SIP [17]	∼3.0	∼5.0	∼4.0	∼$11.0^{\circ }$	∼$16.0^{\circ }$	∼$16.0^{\circ }$

**Table 6:** Knee and hip angle RMSE no bias (top) and CC (bottom) of CKF-3IMU, OSPS, and related literature for free walk.

Joint Angle RMSE (${}^{\circ }$)	Knee Sagittal	Hip Sagittal	Hip Frontal	Hip Transverse
L5S-3IMU	7.6±2.6	6.6±2.7	5.0±2.6	8.6±3.6
L5S-3IMU+D	7.1±2.1	7.5±2.1	5.1±2.3	8.9±3.7
OSPS	5.0±1.8	3.6±1.7	4.1±2.2	11.9±4.3
CKF-3IMU [15]	5.7±2.2	4.4±1.9	5.5±2.6	9.0±3.8
Cloete et al. [37]	8.5±5.0	5.8±3.8	7.3±5.2	7.9±4.9
Hu et al. [38]	4.9±3.5	6.8±3.0	-	-
Tadano et al. [7]	10.1±1.0	7.9±1.0	-	-
**Joint Angle CC**	**Knee Sagittal**	**Hip Sagittal**	**Hip Frontal**	**Hip Transverse**
L5S-3IMU	0.95±0.03	0.87±0.16	0.76±0.18	0.36±0.36
L5S-3IMU+D	0.95±0.03	0.83±0.14	0.72±0.19	0.29±0.37
OSPS	0.97±0.04	0.95±0.06	0.72±0.19	0.26±0.20
CKF-3IMU [15]	0.98±0.03	0.96±0.08	0.73±0.17	0.26±0.39
Cloete et al. [37]	0.89±0.15	0.94±0.08	0.55±0.40	0.54±0.20
Hu et al. [38]	0.95±0.04	0.97±0.04	-	-
Tadano et al. [7]	0.97±0.02	0.98±0.01	-	-

## Abbreviations

The following abbreviations are used in this manuscript:

OMC	Optical Motion Capture
IMC	Inertial Motion Capture
IMU	Inertial Measurement Unit
OSPS	One Sensor per Body Segment
RSC	Reduced-Sensor-Count
KF	Kalman Filter
EKF	Extended Kalman Filter
CEKF	Constrained Extended Kalman Filter
ADL	Activities of Daily Living
TTD	Total Travelled Distance
SOP	Sparse Orientation Poser
SIP	Sparse Inertial Poser

## Appendix A. Derivation of Pelvis-to-Ankle Distance Measurement

This section explains the derivation of the measurement pelvis-to-ankle vector (Equation (46)) as obtained from pelvis-to-ankle distance measurements, ${\overset{˘}{d}}_{k}^{pla}$ and ${\overset{˘}{d}}_{k}^{pra}$, while assuming hinged knee joints and constant body segment lengths. For the sake of brevity, only the left side formulation is shown. The right side (i.e., pelvis to right ankle vector) can be calculated similarly.

First, we solve for an estimated left knee angle, ${\hat{\theta }}_{k}^{lk}$ (Equation (47)), from the measured pelvis to left ankle distance, ${\overset{˘}{d}}_{k}^{pla}$. The pelvis to left ankle vector, $\tau_{m}^{pla}\left({\hat{\mu }}_{k}^{-},\theta_{k}^{lk}\right)$ (Equation (A6)), can be defined as the sum of the mid-pelvis to hip, thigh long axis, and shank long axis vectors.

(A1) $$\begin{array}{c} \tau_{pla}^{}\left({\hat{\mu }}_{k}^{-},\theta_{k}^{lk}\right)=\overset{\psi_{pla}=\mathrm{half}\;\mathrm{pelvis}\;y-\mathrm{axis}\;+\;\mathrm{shank}\;z-\mathrm{axis}}{\overset{︷}{\frac{d^{p}}{2}{\hat{T}}_{k}^{p-}\;i_{y}^{}-d^{ls}{\hat{T}}_{k}^{ls-}\;i_{z}^{}}}+d^{lt}{\hat{T}}_{k}^{ls-}\overset{\mathrm{thigh}\;z-\mathrm{axis}\;\mathrm{in}\;\mathrm{shank}\;\mathrm{frame}}{\overset{︷}{(i_{x}^{}\sin\left(\theta_{k}^{lk}\right)-i_{z}^{}\cos\left(\theta_{k}^{lk})\right)}} \end{array}$$

By definition of ${\left({\overset{˘}{d}}_{k}^{pla}\right)}^{2}$ and expanding $\tau_{m}^{pla}\left({\hat{\mu }}_{k}^{-},\theta_{k}^{lk}\right)$ with Equation (A1), we obtain

(A2) $$\begin{array}{c} \begin{array}{cc} {\left({\overset{˘}{d}}_{k}^{pla}\right)}^{2} & ={\left(\tau_{pla}^{}\left({\hat{\mu }}_{k}^{-},\theta_{k}^{lk}\right)\right)}^{T}\tau_{pla}^{}\left({\hat{\mu }}_{k}^{-},\theta_{k}^{lk}\right)\\ & =\psi_{pla}^{T}\psi_{pla}-2d^{lt}\psi_{pla}^{T}{\hat{T}}_{}^{ls-}\;i_{z}^{}\cos\left(\theta_{k}^{lk}\right)+2d^{lt}\psi_{pla}^{T}{\hat{T}}_{}^{ls-}\;i_{x}^{}\sin\left(\theta_{k}^{lk}\right)+{\left(d^{lt}\right)}^{2} \end{array} \end{array}$$

Equation (A2) can be rearranged in the form of Equation (A3) with α, β, γ as shown in Equation (A4).

(A3) $$\begin{array}{c} \alpha \cos\left(\theta_{k}^{lk}\right)+\beta \sin\left(\theta_{k}^{lk}\right)=\gamma \end{array}$$

(A4) $$\begin{array}{c} \alpha =-2d^{lt}\psi_{pla}^{T}{\hat{T}}_{k}^{ls-}\;i_{z}^{},\;\beta =2d^{lt}\psi_{pla}^{T}{\hat{T}}_{k}^{ls-}\;i_{x}^{},\;\gamma ={\left({\overset{˘}{d}}_{k}^{pla}\right)}^{2}-\psi_{pla}^{T}\psi_{pla}-{\left(d^{lt}\right)}^{2} \end{array}$$

Solving for ${\hat{\theta }}_{k}^{lk}$ from Equation (A3) gives us a quadratic equation with two solutions as shown in Equations (A5) and (47). Between the two solutions, ${\hat{\theta }}_{k}^{lk}$ is set as the ${\hat{\theta }}_{k}^{lk}$ whose value is closer to the current left knee angle estimate from the prediction step. This solution serves as a pseudomeasurement of the knee angle.

(A5) $$\begin{array}{c} {\hat{\theta }}_{k}^{lk}=\cos^{-1}\left(\frac{\alpha \gamma \pm \beta \sqrt{\alpha^{2}+\beta^{2}-\gamma^{2}}}{\alpha^{2}+\beta^{2}}\right) \end{array}$$

Finally, $Z_{pla,k}$, the KF measurement shown in Eqs. (A6) and (46), is the inter-IMU vector between the pelvis and left ankle, calculated using Equation (A1) with input ${\hat{\theta }}_{k}^{lk}$.

(A6) $$\begin{array}{c} Z_{pla,k}=\tau_{m}^{pla}\left({\hat{\mu }}_{k}^{-},{\hat{\theta }}_{k}^{lk}\right) \end{array}$$
